# Hydrazinolysis Products of Selected Sugar Lactones—Crystal Structure and Microbiological Activity

**DOI:** 10.3390/ijms241512114

**Published:** 2023-07-28

**Authors:** Justyna Samaszko-Fiertek, Artur Sikorski, Barbara Dmochowska, Piotr Szweda, Janusz Madaj

**Affiliations:** 1 Faculty of Chemistry, University of Gdansk, Wita Stwosza 63, 80-308 Gdansk, Poland; 2 Department of Pharmaceutical Technology and Biochemistry, Gdansk University of Technology, Gabriela Narutowicza Street 11/12, 80-233 Gdansk, Poland

**Keywords:** sugar hydrazides, hydrazinium ascorbate, TG analysis, crystallography, antimicrobial activity

## Abstract

Commercially available lactones, as well as those synthesized by us, turned out to be good substrates for the synthesis of sugar hydrazides. The exception was L-ascorbic acid, whose hydrazinolysis led to the formation of a hydrazinium salt, not the hydrazide as expected. The structure of all compounds was confirmed by NMR and X-ray analyses. The lower durability of hydrazinium L-ascorbate was additionally confirmed by thermogravimetric tests. All products were tested for biological activity against Gram-negative bacteria strains *Escherichia coli* ATCC 25922 and *Pseudomonas aeruginosa* ATCC 27853 and against Gram-positive *Staphylococcus aureus* ATCC 25923 and *Staphylococcus aureus* ATCC 29213. Their antifungal activity against *Candida albicans* SC5314, *Candida glabrata* DSM 11226 SM 11226, *Candida krusei* DSM 6128, and *Candida parapsilosis* DSM 5784 was also tested. The most interesting results of microbiological activity were obtained for D-gluconic acid hydrazide and hydrazinium L-ascorbate. The results of the latter encourage more extensive testing.

## 1. Introduction

Hydrazides, according to the definition recommended by IUPAC, are compounds derived from oxoacids R*_k_*E(=O)*_l_*(OH)*_m_ *(*l* ≠ 0) by replacing –OH by –NRNR_2_ (R groups are commonly H), as in carbohydrazides, RC(=O)NHNH_2_, sulfonohydrazides, RS(=O)_2_NHNH_2_, and phosphonic dihydrazides, RP(=O)(NHNH_2_)_2_ [1]. Many examples of reactions leading to obtaining the corresponding hydrazide derivatives are described in the literature: from alcohols [2,3,4], from aryl tosylates [5], from arenes [6], from styrenes [7], from aryl halides [8,9,10,11], by alkylation of hydrazine [12,13], and from heteroarylboronic acids [14]. However, still the most commonly used methods of hydrazide synthesis use esters and carbonyl compounds [15,16,17,18].

There are many interesting examples of the use of hydrazides in the literature. One of the first effective antibiotics used in the treatment of *Mycobacterium tuberculosis* was isonidazid (pyridine-4-carboxylic acid hydrazide). It was first synthesized in 1912, but its effectiveness as a drug against tuberculosis was discovered only in the 1950s [19,20]. Some hydrazide derivatives have antiretroviral properties, including HIV-1 [21,22,23]. In addition, the derivatives of isonidazid (*N*′-isopropyl and the *N*′-benzyl) show activity against monoamine oxidase, responsible for the deamination of serotonin and norepinephrine [24]. 

An interesting example of a hydrazide with biological activity is carbidopa hydrazine acid. It affects the rate of dopamine release, prolonging the effect of the drug in the treatment of Parkinson’s disease [25].

Hydrazides are also important substrates for obtaining an extremely interesting group of compounds, which are hydrazide–hydrazones. This is a group of compounds in which alkyl or aryl groups are connected by a bridge -C(=O)-NH-N=CH-. There are many examples of their biological activity [26].

Carbohydrate hydrazides, despite their high potential, are still a little-studied group of compounds. They are obtained mainly from free sugars by the reaction of the carbonyl group of aldoses with hydrazine [27,28,29,30], as derivatives of uronic acids [31], or from D-ribono-1,4-lactone [32].

In this paper, we present the synthesis, structure, and biological activity studies of a selected group of compounds that are products of the reaction of sugar lactones with hydrazine.

## 2. Results and Discussion

**Figure 1:**
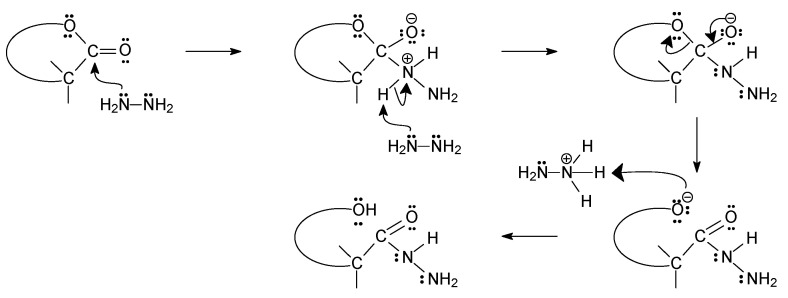
Proposed mechanism for the reaction of sugar lactones with hydrazine.

The lactones used for the reaction with hydrazine were commercially available (D-gluconolactone and L-ascorbic acid) or were obtained from commercially available sugars (D-ribose, 2-deoxy-D-ribose, and D- and L-fucose). For their synthesis, the bromine oxidation method in the presence of potassium carbonate developed for D-ribono-1,4-lactone was used [33]. The indisputable advantage of this method is its simplicity, the possibility of conducting it on a large scale, as well as the high yields and high purity of the obtained products. In the case of synthesized lactones, the size of the ring (γ- or δ-lactones) was not important because after their isolation, they underwent further reaction without the need for purification, and the hydrazides formed were not affected by the size of the lactone ring, as shown in Figure 1 below.

The choice of the configuration of the synthesized hydrazides resulted from the desire to study the wide range of structural systems occurring in natural sugars; therefore, we used three hexoses, two pentoses, and ascorbic acid for the synthesis. Among the hexoses, we used the most common D-glucose and two enantiomers of fucose (6-deoxy galactose). The use of D- and L-fucose allowed us to test the possible impact of the lack of a hydroxyl group at the terminal carbon atom C6 and to compare the activity of the two enantiomers. The studies of D-ribose and 2-deoxy-D-ribose were able to determine the effect of the length of the carbon chain (compared with hexoses) and the lack of a hydroxyl group in the α position to the hydrazide moiety on the structure and biological activity. The choice of ascorbic acid resulted from its known biological activity and the fact that a C=C double bond would be present in the obtained hydrazide. 

**Figure 2:**
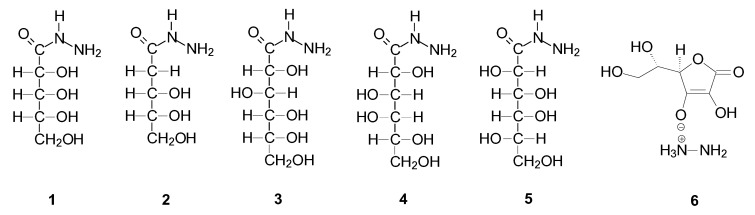
The structures of the obtained products.

In all cases of the synthesis, after some time, we observed the precipitation of a white precipitate from the methanolic lactone and hydrazine solution, which was then recrystallized from a mixture of methyl alcohol and water. The obtained crystal deposits showed high purity, which was confirmed by the results of elemental analysis, and the crystals were of such good quality that it was possible to determine the crystal structure for all of them. First, we confirmed the structure of all the products by performing NMR spectroscopic analyses. Full characterization of all compounds is presented in Table S4 (Supplementary Materials). Although the synthesis of D-gluconic and D-ribonic acid hydrazides has been previously described in the literature [34], we decided to include it in this article since we were unable to find their full characteristics in the literature. The structures of all obtained compounds are shown in Figure 2.

**Figure 3:**
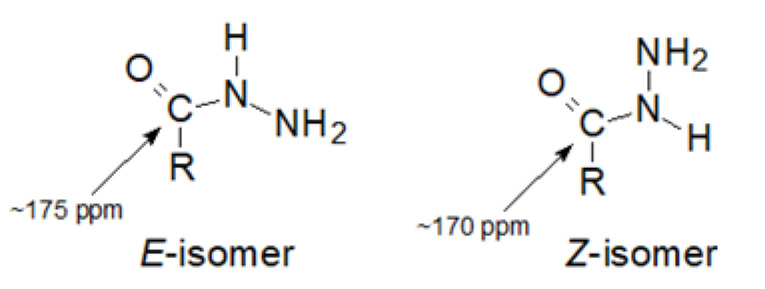
The Z/E isomerism of the hydrazide moiety.

Looking at the chemical shift values of the C1 carbon atom in compounds **1–5**, it can be seen that they range between 172.51 and 173.91 ppm (full NMR spectra are shown in Figures S1–S24 in Supplementary Materials). Such a value of these shifts, if based on the literature data [35], may indicate that the E-isomer predominates in the aqueous solution. As shown by the authors’ studies using modern NMR techniques of the Z/E isomerism of hydrazides, the chemical shift of the carbonyl carbon atom in the Z-isomer was below or approx. 170 ppm, while in the E-isomer it was approx. 175 ppm (Figure 3).

**Figure 4:**
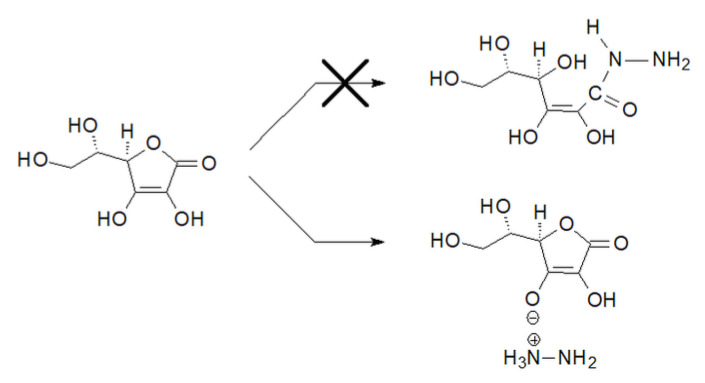
Reaction of L-ascorbic acid with hydrazine.

In the case of compound **6**, the value of the carbon C1 chemical shift reached an even higher value of 177.51 ppm. More surprising, however, was the value of the chemical shift in this compound of the C3 carbon atom, which was 175.59 ppm. Such values indicated that it was not ascorbic acid hydrazide. Such a high value also did not correspond to the chemical shift of the C3 carbon atom in the substrate, which was 155.22 ppm [36]. Studies of the literature data have shown that such high values of chemical shifts of carbon atoms C1 and C3 are close to the value of shifts of these carbon atoms in sodium ascorbate [37]. The results obtained in further studies explained such a high value of the chemical shift of the C3 carbon atom. It was the result of the transfer of a proton from the C3-OH group to the hydrazine molecule and the formation of an appropriate anion. L-Ascorbic acid turned out to be such a strong acid that instead of the assumed nucleophilic attack of the hydrazine nitrogen atom on the carbonyl carbon atom, the hydrazine molecule was protonated as a result of proton transfer from the enol group at the carbon atom C3, resulting in the formation of a hydrazinium salt (Figure 4).

In addition, the lower stability of compound **6** in comparison with derivatives **1–5** could indicate its structure as being other than hydrazide. The positive mode ESI MS spectrum showed only the *m*/*z* 174.7 ion, while the negative mode ESI MS contained the two main ions *m*/*z* 176.9 and 209.0 (see Figures S25–S27 in Supplementary Materials for more details). Such a spectrum is often characteristic of ionic compounds, and while the *m*/*z* 209.0 ion was not a surprise [M + 1], the ions 174.7 and 176.9 closely resembled the ions characteristic of ascorbic acid [M 176.1]. In addition, an analysis of the positive MS-MS spectrum showed that the fragmentation of the *m*/*z* 176.9 ion was very similar to that of ascorbic acid [38]. There were fragmentation ions *m*/*z* 158.9 [176.9–1H_2_O] and 141.0 [176.9–2H_2_O]. All these results showed that compound 6 was not likely to be a hydrazide, and its behavior was more indicative of some derivative of ascorbic acid. Its low stability (24 h in an aqueous solution and approx. 30 days in a crystalline form at −20 °C) prompted us to perform a thermogravimetric analysis for it (Figure S28 Supplementary Materials). Up to a temperature of approx. 140 °C, compound **6** showed high thermal stability and did not melt. It began to decompose at approx. 146 °C, unlike L-ascorbic acid, which begins to decompose at approx. 191 °C (Merck Index, 14th ed.). During the thermal decomposition of the analyzed compound, a mixture of gases was observed, including H_2_O, CO_2_, CO, and ammonia. It was a multi-stage process, but due to the speed of the processes, it was not possible to determine the individual stages of the sample’s decomposition or to determine the exact mass losses. Above the temperature of 152 °C, a significant increase in the quantity of gaseous products was observed, and their structure was determined by the recorded IR spectra. At the wavenumber values of 932.72 cm^−1^ and 961.83 cm^−1^, absorbance signals characteristic of ammonia or its derivatives (N-H) appeared, which confirmed that this compound was formed as a result of the reaction of hydrazine and ascorbic acid. In addition, at wavenumbers 2155, 2121.8 cm^−1^, and approx. 2350 cm^−1^, signals corresponding to νC=O vibrations appeared, indicating the formation of CO and CO_2_ (Figure S29A, see Supplementary Materials). With an increase in the temperature of approx. 7.5 °C, there was an increase in the release of water vapor (H_2_O). This confirmed the presence of signals on the IR spectrum at the wavenumber values of 3400–4000 cm^−1^ and 1500–1600 cm^−1^, corresponding to νO-H vibrations. In contrast to the TG analysis curve for pure L-ascorbic acid, it was difficult to confirm the formation of HCOOH in the released gases due to the possibility of their being masked by the presence of a significant amount of H_2_O as a result of dehydration. At a temperature of approx. 272 °C, there was a significant decrease in H_2_O and NH_3_ emissions (Figure S29B, see Supplementary Materials). In the final stage of decomposition, CO_2_ was released in the largest quantity, which is confirmed by the IR spectra.

### 2.1. Crystal Structure and Analysis of Intermolecular Interactions

**Figure 5:**
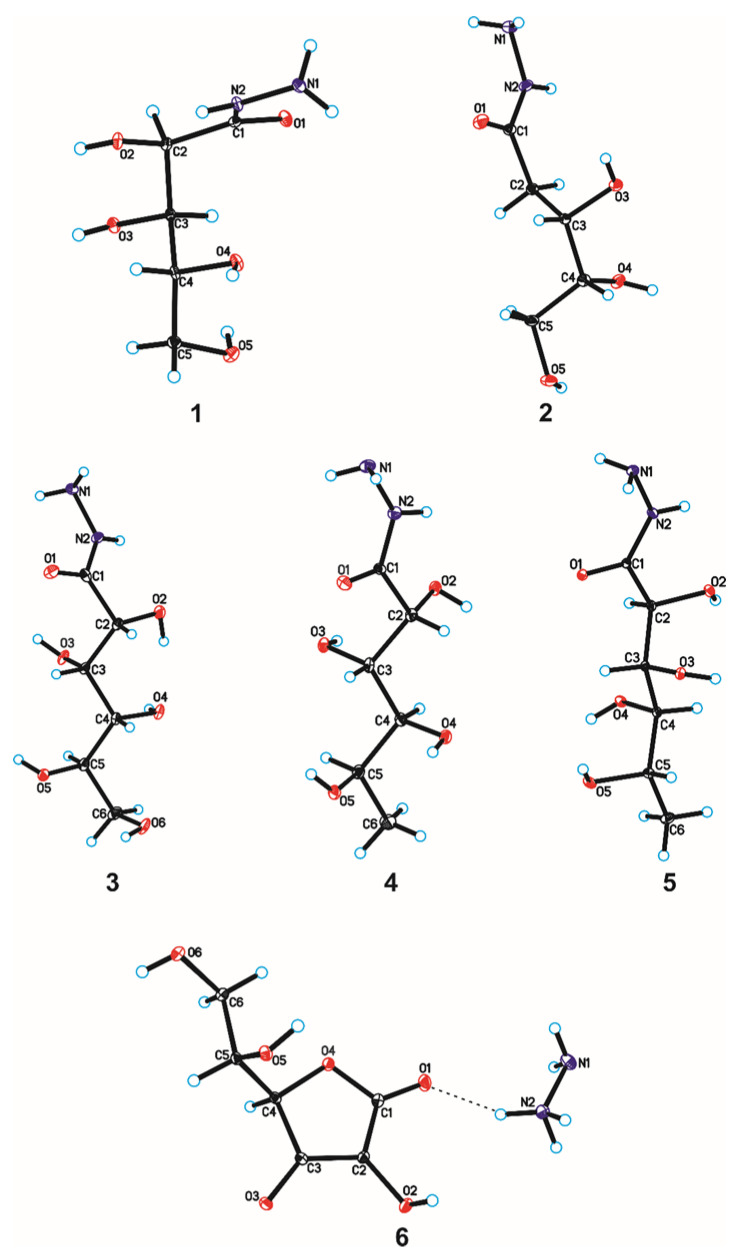
Molecular structures of compounds **1–6** with the atom-labelling scheme. Displacement ellipsoids are drawn at the 15% probability level, and H atoms are shown as small spheres of arbitrary radius (hydrogen bonds are represented by dashed line).

**Figure 6:**
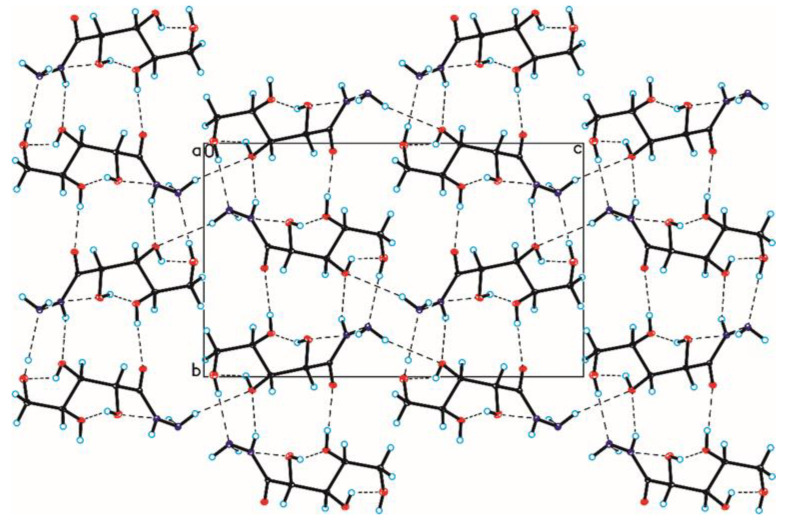
Crystal packing of compound 1 viewed along the a-axis (hydrogen bonds are represented by dashed lines).

**Figure 7:**
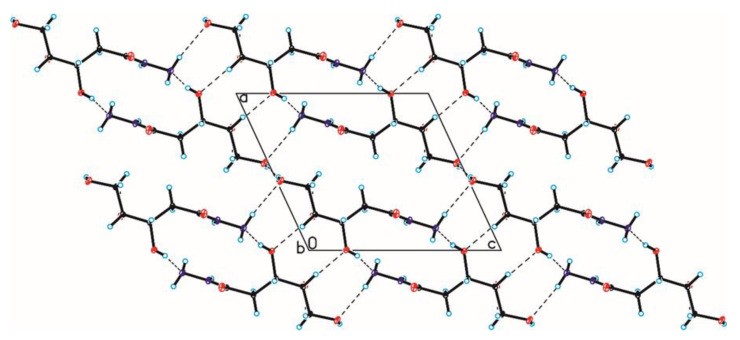
Crystal packing of compound 2 viewed along the b-axis (hydrogen bonds are represented by dashed lines).

**Figure 8:**
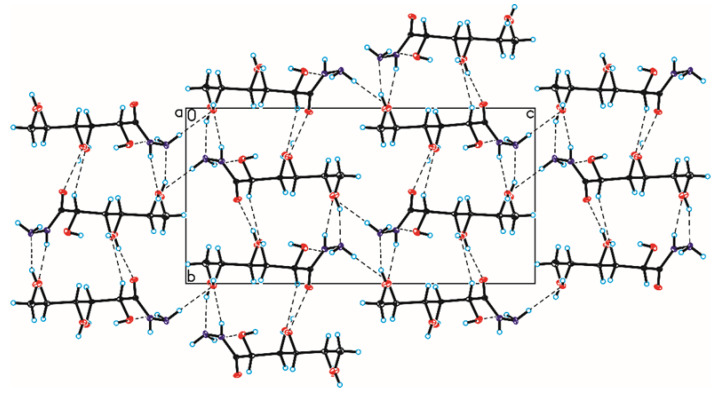
Crystal packing of compound 3 viewed along the a-axis (hydrogen bonds are represented by dashed lines).

**Figure 9:**
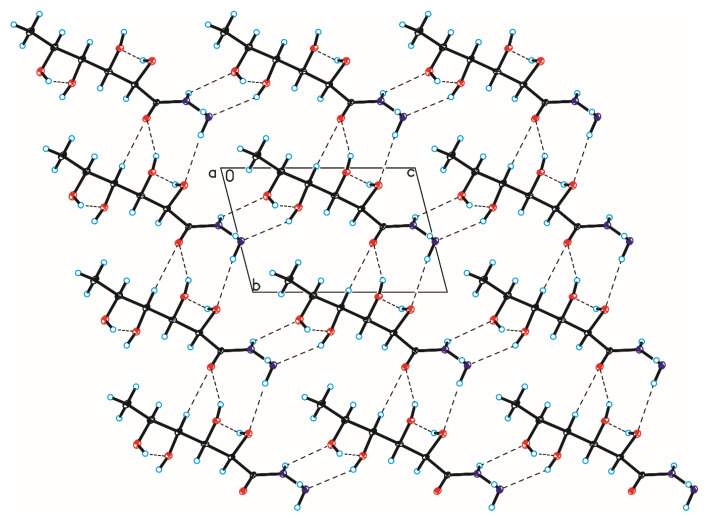
Crystal packing of compound 4 viewed along the a-axis (hydrogen bonds are represented by dashed lines).

**Figure 10:**
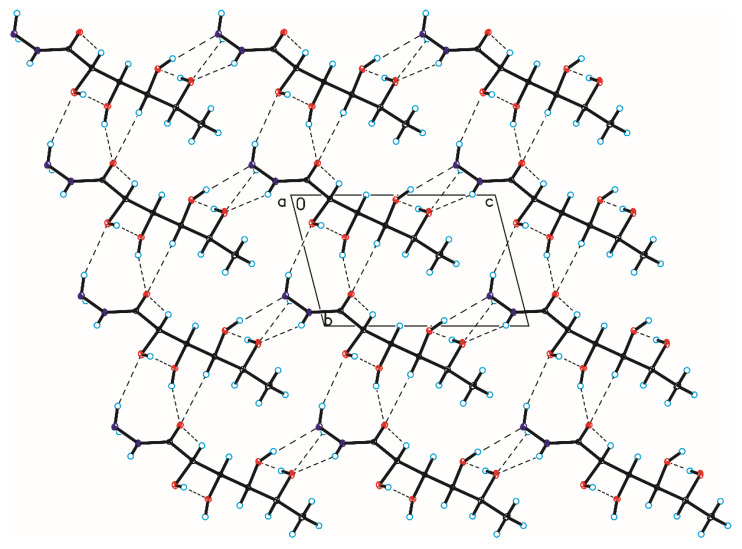
Crystal packing of compound 5 viewed along the a-axis (hydrogen bonds are represented by dashed lines).

**Figure 11:**
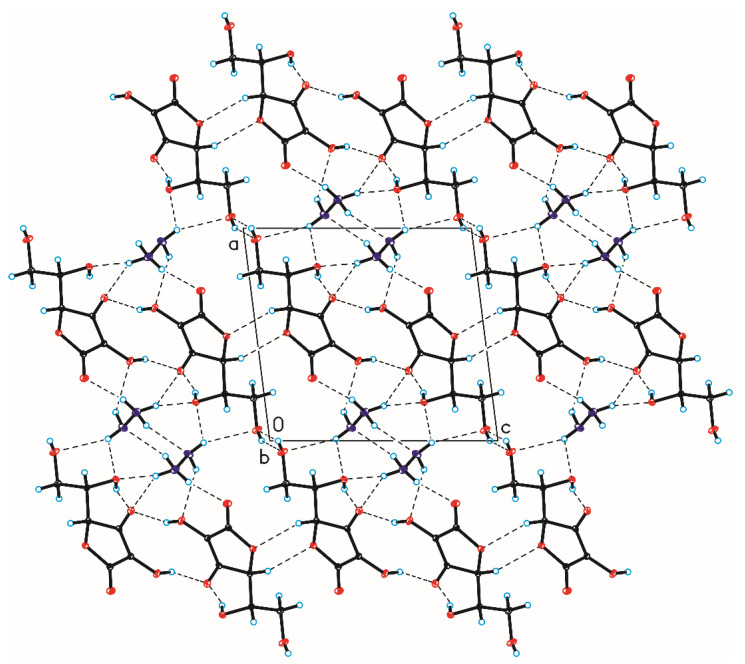
Crystal packing of compound 6 viewed along the b-axis (hydrogen bonds are represented by dashed lines).

The crystallographic structures of compounds **1–6** with the numbering of atoms are shown in Figure 5. Full crystal data and structure refinement for compounds **1–6** are included in Table 1. All obtained hydrazides in the crystal lattice existed as Z isomers. Hydrazides **2–5** with a chain structure adopted a similar zigzag conformation in the crystal lattice. The situation was different in the case of D-ribonic acid hydrazide (**1**), in which the molecule was rotated around the C2-C3 bond. This may have been due to the difference in the arrangement of the hydroxyl groups at the C2 and C3 atoms in the hydrazide with the D-ribo, D-gluco, and D- or L-fuco configurations. Due to the lack of a hydroxyl group at the C2 carbon atom, this was not observed in the case of 2-deoxy-D-ribonic acid hydrazide. Such rotation enabled the formation of an intramolecular hydrogen bond between the hydrogen atom of the hydroxyl group on the C2 carbon atom and the oxygen atom of the hydroxyl group on the C4 carbon atom (C4-O-H···O4). This bond is shown in Figure 6, and its data are included in Table S2 (Supplementary Materials). All compounds formed a large number of hydrogen bonds in the crystal lattice, shown in Figure 6, Figure 7, Figure 8, Figure 9, Figure 10 and Figure 11, and the full geometry of these bonds is included in Tables S2 and S3 (Supplementary Materials). Compound **6** contained a nearly planar five-membered ring in which the torsion angle C1-C2-C3-C4 was 1.2(4) degrees.

**Figure 12:**
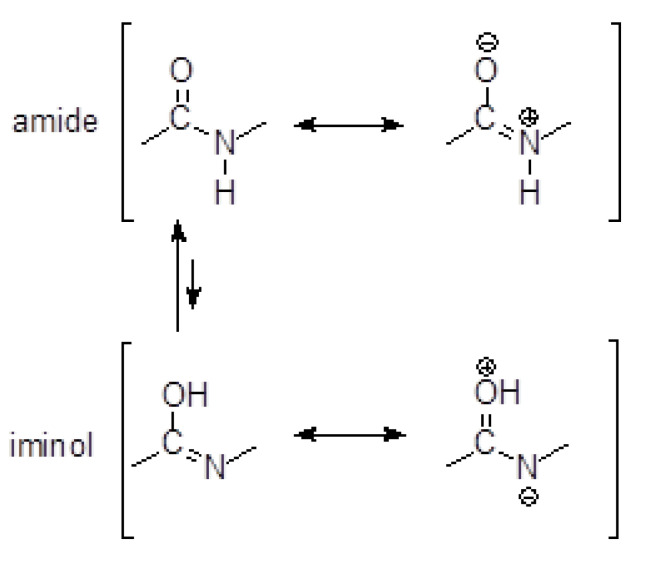
Amide–iminol tautomerism.

Due to the amide–iminol tautomerism shown in Figure 12, hydrazides resemble a peptide bond in structure. 

The parameters of selected bond lengths and angles of hydrazides **1–5** are presented in Table 2. 

Their analysis showed that, similarly to the peptide bond, the C=O bond was elongated in relation to the analogous bond in carboxylic acids, which was 1.20 Å. As in the case of the peptide bond, the C-N bond partially acquired the character of a double bond, which resulted in its significant shortening. A typical single C-N bond has a length of 1.47 Å [39], while in the analyzed hydrazides, it did not exceed 1.35 Å. In addition, the N-N bond in the crystals of compounds **1–5** was shorter than that in the hydrazine crystal lattice, which was 1.46 Å [40]. Perhaps this was due to the interaction of the non-bonding electron pair of the terminal nitrogen atom with the delocalized pair in the C-N bond. As in the case of the peptide bond, the O-C-N-H atoms were located almost in one plane.

Compound **6** as a derivative of L-ascorbic acid had a cyclic structure. Selected bond lengths of compound **6** compared with ascorbic acid are shown in Table 3.

The analysis of the data contained in Table 3 showed that as a result of delocalization of electrons in the ascorbate anion, the C1=O and C2-C3 bonds were elongated compared with the undissociated L-ascorbic acid molecule. On the other hand, the C3-O bond was significantly shortened, becoming more of a double bond. This may explain the above-described difference in the chemical shift of the C3 carbon atom.

### 2.2. Microbiological Testing

The conducted research revealed some important differences in the antibacterial as well as the antifungal potential of the synthesized products. The growth of both the reference strains of staphylococci was inhibited only by compound **6** (hydrazinium L-ascorbate) at its highest concentration (512 µg/mL). Gram-negative bacteria, particularly E. coli, exhibited an importantly higher level of susceptibility. The MIC values (Table 4) for this strain were in the range of concentrations from 64 µg/mL (compound 3) to 512 µg/mL (compound **2**). P. aeruginosa ATCC 27853 exhibited the highest sensitivity to D-rybonic acid hydrazide (**1**) (MIC = 128 µg/mL) and was most resistant (MIC = 512 µg/mL) to 2-deoxy-D-derybonic acid hydrazide (**2**), whereas other compounds inhibited the growth of this strain at the concentration 256 µg/mL. 

The most interesting observation from this part of the study devoted to the analysis of the antifungal activity of the synthesized hydrazides was an important dependence of their activity on the composition of the growth medium used for the assay (Table 5). Interestingly, all strains tested exhibited the highest level of susceptibility in the RPMI medium not supplemented with glucose. This medium contained glucose at the final concentration of 2.0 g/L, whilst the two other media contained 2.0% (*w/w*) of glucose, and the concentration of this sugar was the only difference between the two RPMI media. The MIC values for the most sensitive C. albicans strain in RPMI+GLC, RPMI-GLC and minimal YNB medium were in the ranges 32–128, 32–64, and 128->512 µg/mL, respectively. The highest inhibitory activity against this strain was shown by hydrazinium L-ascorbate (**6**) and D-gluconic acid hydrazide (3), with MIC values of 32, 32, and 128 and 32, 32, and 256 µg/mL respectively. Among the compounds, the highest activity was exhibited by D-gluconic acid hydrazide (**3**)—a derivative of glucose. Up to a concentration of 512 µg/mL, this hydrazide was able to inhibit the growth of all strains tested in all media except C. glabra and C. krusei, which were grown in minimal YNB medium (containing 2.0% of glucose). Compound **2** exhibited the lowest antifungal potential and was able to inhibit the growth of only the C. albicans strain in both RPMI media. In the RPMI medium not supplemented with glucose, compounds **1**, **4**, **5**, and **6** effectively inhibited the growth of C. glabra, C. krusei, and C. parapsilosis at concentrations of 256 or 512 µg/mL. Considering the tested range of concentrations, none of the synthesized compounds was able to inhibit the growth of the C. glabra and C. krusei in the YNB medium, whilst compounds **1**, **3**, and **4** inhibited the growth of C. parapsilosis in this medium at the highest investigated concentration—512 µg/mL. 

## 3. Materials and Methods

### 3.1. General Section

All sugar substrates: D-ribose, 2-deoxy-D-ribose, D-glucono-1,5-lactone, D-fucose, L-fucose, and L-ascorbic acid were purchased from Biosynth Ltd. (Compton, UK).

### 3.2. NMR Measurements

All measurements were carried out on a Bruker 500 MHz spectrometer. All spectra were recorded at controlled temperature of 298 K using TXI inverse probe. Obtained spectra were processed and analyzed with the usage of TopSpin 3.2 (Bruker BioSpin GmbH, Mannheim, Germany) software.

### 3.3. MS Spectrometry

MS spectra with ESI ionization were recorded using a Bruker Daltonics HCT Ultra spectrometer with an ion trap analyzer.

### 3.4. TG Analysis

Thermogravimetric analysis was performed using the TG209 thermobalance by Netzsch coupled with FT-IR, which enabled infrared (MIR) analysis of gaseous thermal decomposition products of the tested sample. High sensitivity of both thermogravimetric and infrared measurements was ensured by the liquid-nitrogen-cooled MCT detector. A sample of 5726 mg was analyzed in the temperature range of 28–450 °C, with a temperature increase of 10 °C per min in an argon atmosphere.

### 3.5. Elemental Analysis

Elemental analysis of the samples was performed using the Elementarny Vario El Cube CHNS analyzer by Elementar (Elementar Analysensysteme GmbH, Hesse, Germany). Measurements were taken twice (full results are in Table S1 in Supplementary Materials), and their values were averaged.

### 3.6. Polarimetry

Polarimetric measurements for both enantiomers of fuconic acid hydrazides (compound **4** and **5**) were performed in water using the ISS REPo-1 Portable Refracto-Polarimeter by ATAGO (ATAGO Co. Ltd., Tokyo, Japan).

### 3.7. Single-Crystal X-ray Diffraction

Single-crystal X-ray diffraction data were collected on an Oxford Diffraction Gemini R ULTRA Ruby CCD diffractometer with MoKα (λ = 0.71073 Å) radiation at T = 295(2) K (Table 1). The lattice parameters were obtained by least-squares fit to the optimized setting angles of the reflections collected by means of CrysAlis CCD [39]. Data were reduced using CrysAlis RED software (Version 1.171.36.24) [42] by and applying multi-scan absorption corrections. The structural resolution procedure was carried out using the SHELX package [43]. The structures were solved with direct methods that carried out refinements by full-matrix least-squares on F2 using the SHELXL-2017/1 program [43]. All H-atoms bound to O/N-atoms were located on a difference Fourier map and refined freely with Uiso(H) = 1.5/1.2Ueq(O/N). All H-atoms bound to C-atoms were placed geometrically and refined using a riding model with d(C–H) = 0.97–0.98 Å and Uiso(H) = 1.2Ueq(C) or with d(C–H) = 0.96 Å and Uiso(H) = 1.5Ueq(C) for the methyl groups. All interactions were calculated using the PLATON program [44]. The following programs were used to prepare the molecular graphics: ORTEPII [45], PLUTO-78 [46], and Mercury [47]. Full crystallographic details of the structures reported in this paper have been deposited with the Cambridge Crystallographic Data Centre (deposition No. CCDC 2262959, CCDC 2262961, CCDC 2262964, CCDC 2262963, CCDC 2262960, and CCDC 2262962 for compounds **1–6,** respectively), and they may be obtained from www: http://www.ccdc.cam.ac.uk (accessed on 2 July 2023), e-mail: deposit@ccdc.cam.ac.uk, or The Director, CCDC, 12 Union Road, Cambridge, CB2 1EZ, UK.

### 3.8. Antimicrobial Activity

The antimicrobial potential of the synthesized hydrazides was evaluated against two reference strains of Gram-negative bacteria (Escherichia coli ATCC 25922 and Pseudomonas aeruginosa ATCC 27853), two reference strains of Gram-positive staphylococci (Staphylococcus aureus ATCC 25923 and Staphylococcus aureus ATCC 29213), and four reference strains of pathogenic yeasts of the genus *Candida* spp. (Candida albicans SC5314, Candida glabrata DSM 11226, Candida krusei DSM 6128, and Candida parapsilosis DSM 5784). The assays were performed using a serial, twofold dilution method in 96-well microtiter plates under conditions recommended by the Clinical and Laboratory Standards Institute (CLSI, Pittsburgh, PA, USA). The aim of this procedure was the determination of the MIC parameter (Minimum Inhibitory Concentration)—the minimum concentration of a tested agent capable of inhibiting the growth of a specified strain of microorganism. In the case of bacterial strains, the assay was performed in Mueller–Hinton Broth (MHB) medium, and three different media were used for assessment of the antifungal potential of the synthesized compounds, namely RPMI (RPMI—10.4 g/L; Glucose 18 g/L, MOPS—35 g/L; this medium contained glucose at a final concentration of 2% (*w/v*), pH 7.0), RPMI not supplemented with glucose (RPMI—10,4 g/L; MOPS—35 g/L; this medium contained glucose at final concentration of 0.2% (*w/v*), pH 7.0), and YNB (Yeast Nitrogen Base with (NH4)2SO4—6.8 g/L, Glucose 20 g/L; this medium contained glucose at final concentration of 2% (*w/v*), pH of this medium was not adjusted). All three media dedicated to yeasts were filter-sterilized, whilst the MHB medium was sterilized in an autoclave. The amount of 1024 µg/mL solutions of all the synthesized hydrazides were prepared in all the above-mentioned sterile media. In the next step, the serial, twofold dilutions of the tested agents (over a range of concentrations from 1024.0 to 2.0 µg/mL) were prepared in the rows of 96-well microtitration plates, with a final volume of 100 μL of the appropriate medium. The bacterial strains were grown on the Mueller–Hinton Agar (MHA) for 18–24 h at 37 °C, and yeasts strains were cultivated on the YPD agar in the same conditions. A small amount of the biomass of the culture of each strain of microorganisms was suspended in the sterile PBS (phosphate buffered saline, pH 7.4 at 25 °C, purchased from Sigma) solution to obtain an optical density OD600 = 0.13 (for bacteria—equal to the cells concentration of approximately 1 × 108 CFU/mL) and OD660 = 0.10 (for yeasts—equal to the cells concentration of approximately 1 × 10^6^ CFU/mL). The obtained suspensions of the bacterial strains were then diluted 1:100 (*v/v*) in the MHB2 medium, whilst yeasts’ suspensions were diluted 1:50 (*v/v*) in the appropriate medium. Then, 100 μL of the cells’ suspension was finally loaded into the wells of plates prepared in advance, which contained 100 μL of twofold dilutions of the tested hydrazides (the final concentration of the bacterial cells in all wells was approximately 5 × 10^5^ CFU/mL and 1 × 10^4^ CFU/mL for yeasts). A positive growth control of each strain (both bacteria and yeasts) was prepared in the wells without the tested substances. A negative control containing only the media was included in each assay. Microtiter plates were incubated at 37 °C for 24 h. Following the incubation period, the determination of the MIC values of the tested agents was carried out by measuring the absorbance at 531 nm using a Victor3 microplate reader (Perkin Elmer, Inc., Waltham, MA, USA). The lowest concentration of the agent causing inhibition of growth equal to or greater than 90% (MIC90) of the growth control was taken as the MIC value. Each test was repeated three times.

### 3.9. General Procedure for the Preparation of Lactones

The appropriate monosaccharide was dissolved in water and K_2_CO_3_ was added. The mixture was cooled to 0 °C, and Br_2_ was added dropwise while stirring. After one hour, the temperature was allowed to rise to room temperature, and the mixture was further stirred for 24 h. Then, the solution was acidified with formic acid to a pH of approx. 3.5, and the volatile components were then evaporated under reduced pressure not exceeding the bath temperature of 50 °C. The residue was extracted three times with ethanol. The combined extracts were filtered and concentrated. The obtained crude lactone was used for the synthesis of hydrazide without further purification.

D-Ribono-1,4-lactone—D-Ribose (8.00 g, 53.3 mM) was dissolved in H_2_O (70 mL), and K_2_CO_3_ was added (8.80 g, 63.7 mM). After cooling to 0 °C, Br_2_ was added dropwise (3.0 mL, 58.5 mM); 8.26 g of crude lactone was obtained.

2-Deoksy-D-ribono-1.4-lactone—2-Deoxy-D-ribose (5.00 g, 37.9 mM) was dissolved in H2O (50 mL), and K2CO3 was added (5.50 g, 40.0 mM). After cooling to 0 °C, Br_2_ was added dropwise (2.0 mL, 39.0 mM); 5.23 g of crude lactone was obtained.

D-Fuconolactone—D-Fucose (3.00 g, 18.3 mM) was dissolved in H_2_O (40 mL), and K_2_CO_3_ was added (3.10 g, 22.4 mM). After cooling to 0 °C, Br_2_ was added dropwise (1.0 mL, 19.5 mM); 3.3 g of crude lactone was obtained.

L-Fuconolactone—L-Fucose (3.00 g, 18.3 mM) was dissolved in H_2_O (40 mL), and K_2_CO_3_ was added (3.10 g, 22.4 mM). After cooling to 0 °C, Br_2_ was added dropwise (1.0 mL, 19.5 mM); 3.5 g of crude lactone was obtained.

### 3.10. General Procedure for the Preparation of Hydrazides

The appropriate lactone was dissolved in methanol and mixed in a glycerin bath at 65 °C. To this mixture was added hydrazine monohydrate, and the mixture was stirred at that temperature for another hour. The mixture then slowly reached room temperature and was stirred for a further 24 h. At this time, the white precipitate was filtered off and recrystallized from a mixture of methanol and water 70:30 (*v/v*).

D-Ribonic acid hydrazide (1)—Crude D-ribono-1,4-lactone (8.26 g) was dissolved in MeOH (70 mL), and hydrazine monohydride was added (2.6 mL, 52.0 mM). Pure hydrazide was obtained after recrystallization (4.47 g, yield after two steps 46.6%, mp. 145.7–146.9 °C, lit. 150 °C [34]). Elemental analysis data: Analyzed Calc. for C_5_H_12_N_2_O_5_: C, 33.34; H, 6.67; N, 15.6. Found: C, 33.34; H, 6.630; N, 15.47. NMR: ^1^H NMR (D_2_O, 500 MHz): 4.32 (d, 1 H, J_1,2_ = 3.72 Hz, H-2), 3,86 (dd, 1 H, J_2,3_ = 3.67 Hz, H-3), 3.79–3.76 (m, 1 H, H-4), 3.74 (dd, 1 H, J_4,5_ = 6.32 Hz, H-5), 3.58 (dd, 1 H, J_5,5′_ = 11.83 Hz, H-5′); ^13^C NMR (D_2_O, 125 MHz): 172.51 C1, 72.66 C3, 72.13 C2, 70.94 C4, 62.83 C5.

2-Deoxy-D-ribonic acid hydrazide (2)—Crude 2-deoxy-D-ribono-1,4-lactone (5.23 g) was dissolved in MeOH (50 mL), and hydrazine monohydride was added (1.9 mL, 38.0 mM). Pure hydrazide was obtained after recrystallization (4.30 g, yield after two steps 70.2%, mp. 136.6-137.4 °C). Elemental analysis data: Analyzed Calc. for C_5_H_12_N_2_O_4_: C, 36.59; H, 7.32; N, 17.07. Found: C, 36.66; H, 7.257; N, 17.04. NMR: ^1^H NMR (D_2_O, 500 MHz): 3.96 (m, 1 H, H-4), 3.69 (dd, 1 H, J_2,2_ = 10.92 Hz, J_2,3_ = 2.12 Hz, H-2), 3.56 (m, 2 H, H-2′, H-3), 2.51 (dd, 1 H, J_4,5_ = 2.82 Hz, J_5,5′_ = 14.63 Hz, H-5), 2.28 (dd, 1 H, J_4,5′_ = 9.80 Hz, H-5′); ^13^C NMR (D_2_O, 125 MHz): 172.92 C1, 74.26 C3, 68.77 C4, 62.38 C5, 37.47 C2.

D-Gluconic acid hydrazide (3)—Commercially available δ-D-gluconolactone (7.00 g, 39.3 mM) was dissolved in MeOH (70 mL), and hydrazine monohydride was added (2.0 mL, 40.0 mM). Pure hydrazide was obtained after recrystallization (4.30 g, yield 89.3%, mp. 143.5–144.5 °C, lit. 142 °C [34]). Elemental analysis data: Analyzed Calc. for C_6_H_14_N_2_O_6_: C, 34.29; H, 6.67; N, 13.33. Found: C, 34.31; H, 6.628; N, 13.22. NMR: ^1^H NMR (D_2_O, 500 MHz): 4.30 (dd, 1 H, J_1,2_ = 4.17 Hz, H-2), 4.05–4.01 (m, 1 H, H-3), 3.79–3.74 (m, 1 H, H-6), 3.72–3.67 (m, 1 H, H-5), 3.67–3.63 (m, 1 H, H-4), 3.62–3.57 (m, 1 H, H-6′); ^13^C NMR (D_2_O, 125 MHz): 172.65 C1, 72.85 C2, 71.74 C4, 71.05 C5, 70.39 C3, 62.63 C6.

D-Fuconic acid hydrazide (4) –Crude D-fuconolactone (3.3 g) was dissolved in MeOH (50 mL), and hydrazine monohydride was added (1.00 mL, 20.0 mM). Pure hydrazide was obtained after recrystallization (1.86 g, yield after two steps 52.4%, decomposition above 191 °C). Specific rotation [α]20D=+15°. Elemental analysis data: Analyzed Calc. for C_6_H_14_N_2_O_5_: C, 37.11; H, 7.22; N, 14.43. Found: C, 37.02; H, 7.263; N, 14.36. NMR: ^1^H NMR (D_2_O, 500 MHz): 4.45 (s, 1 H, H-2), 4.04 (d, 1 H, J_5,6_ = 6.57 Hz, H-5), 3.91 (d, 1 H, J_3,4_ = 9.48 Hz, H-3), 3.43 (d, 1 H, H-4), 1.20 (d, 3 H, H-6); ^13^C NMR (D_2_O, 125 MHz): 173.92 C1, 72.51 C4, 71.11 C3, 70.86 C2, 65.82 C5, 18.69 C6.

L-Fuconic acid hydrazide (5) –Crude L-fuconolactone (3.5 g) was dissolved in MeOH (50 mL), and hydrazine monohydride was added (1.00 mL, 20.0 mM). Pure hydrazide was obtained after recrystallization (1.51 g, yield after two steps 42.5%, decomposition above 191 °C). Specific rotation [α]20D=−15°. Elemental analysis data: Analyzed Calc. for C_6_H_14_N_2_O_5_: C, 37.11; H, 7.22; N, 14.43. Found: C, 37.08; H, 7.259; N, 14.38. NMR: ^1^H NMR (D_2_O, 500 MHz): 4.45 (s, 1 H, H-2), 4.04 (d, 1 H, J_5,6_ = 6.57 Hz, H-5), 3.91 (d, 1 H, J_3,4_ = 9.48 Hz, H-3), 3.43 (d, 1 H, H-4), 1.20 (d, 3 H, H-6); ^13^C NMR (D_2_O, 125 MHz): 173.92 C1, 72.51 C4, 71.11 C3, 70.86 C2, 65.82 C5, 18.69 C6.

Hydrazinium L-ascorbate (6)—Commercially available L-ascorbic acid (5.00 g, 28.4 mM) was dissolved in MeOH (70 mL), and hydrazine monohydride was added (1.42 mL, 28.4 mM). Pure hydrazide was obtained after recrystallization (4.38 g, yield 74.2%, decomposition above 140 °C). Elemental analysis data: Analyzed Calc. for C_6_H_12_N_2_O_6_: C, 34.62; H, 5.77; N, 13.46. Found: C, 34.67; H, 5.746; N, 13.46. NMR: ^1^H NMR (D_2_O, 500 MHz): 4.42 (d, 1 H, J_4,5_ = 1.83 Hz, H-4), 3.92 (m, 1 H, H-5), 3.65 (m, 2 H, H-6 and H-6′); ^13^C (D_2_O, 125 MHz): 177.51 C1, 175.59 C3, 113.09 C2, 78.35 C4, 59.52 C5, 62.52 C6.

## 4. Conclusions

Sugar lactones, both commercially available and those synthesized by us by oxidation of appropriate monosaccharides with bromine, turned out to be good substrates for obtaining sugar hydrazides. At the same time, L-ascorbic acid, which is also a lactone, turned out to be such a strong acid whose reaction with hydrazine led to the formation of hydrazinium L-ascorbate (compound **6**), and not, as initially assumed, to the corresponding hydrazide. We were able to obtain all products of hydrazinolysis in a crystalline form and perform X-ray crystallographic measurements on them. The results of these analyses showed that all obtained hydrazides had the *Z*-isomer structure in the crystal lattice. Moreover, the crystallographic results completely confirmed the structure of hydrazinium ascorbate for the reaction product of L-ascorbic acid with hydrazine.

Summing up, the results of the microbiological tests performed seem to suggest that the lack of a hydroxyl group at the C-2 carbon atom (compound **2**) significantly affected the activity of the compound. Such a strong effect was not observed in the absence of such a group on the C-6 carbon atom (compounds **4** and **5**). Additionally, for these compounds, which are enantiomers, no significant difference in activity was observed. In addition, based on the obtained results, it can be concluded that the length of the carbon chain may have affected the microbial activity (five-carbon in compound **2** and six-carbon in hydrazides **3**, **4**, and **5**). Compound **6** (hydrazinium L-ascorbate) should be considered separately; its results were so interesting that it seems worth subjecting it to a broader study of biological activity.

## Figures and Tables

**Table 1:** Crystal data and structure refinement for compounds **1–6**.

Compound	1	2	3	4	5	6
Chemical formula	C_5_H_12_N_2_O_5_	C_5_H_12_N_2_O_4_	C_6_H_14_N_2_O_6_	C_6_H_14_N_2_O_5_	C_6_H_14_N_2_O_5_	C_6_H_12_N_2_O_6_
FW/g·mol^−1^	180.17	164.17	210.19	194.19	194.19	208.18
Crystal system	orthorhombic	monoclinic	orthorhombic	triclinic	triclinic	monoclinic
Space group	*P*2_1_2_1_2_1_	*P*2_1_	*P*2_1_2_1_2_1_	*P*1	*P*1	*P*2_1_
*a*/Å	5.2059(2)	8.777(2)	5.6086(7)	4.7524(12)	4.7391(6)	8.9247(12)
*b*/Å	9.4219(5)	4.8499(6)	9.1664(12)	5.7522(14)	5.7382(9)	5.1153(7)
*c*/Å	15.3276(9)	9.719(3)	18.223(3)	8.6708(17)	8.6287(15)	9.4937(14)
*α*/°	90	90	90	72.83(2)	72.967(14)	90
*β*/°	90	114.87(3)	90	76.43(2)	76.462(13)	97.139(12)
*γ*/°	90	90	90	75.41(2)	75.323(12)	90
*V*/Å^3^	751.81(7)	375.33(17)	936.8(2)	215.85(9)	213.77(6)	430.05(10)
*Z*	4	2	4	1	1	2
*T*/K	293(2)	293(2)	293(2)	293(2)	293(2)	293(2)
*λ*_Mo_/Å	0.71073	0.71073	0.71073	0.71073	0.71073	0.71073
*ρ_calc_*/g·cm^−3^	1.592	1.453	1.490	1.494	1.508	1.608
µ/mm^−1^	0.142	0.125	0.133	0.129	0.131	0.144
*F(000)*	384	176	448	104	104	220
*θ* range/°	3.43–25.00	4.10–25.00	3.80–25.00	3.78–25.00	3.79–25.00	3.35–25.00
Completeness of *θ*/%	99.8	99.6	99.7	99.9	99.9	99.6
Reflections collected	4849	2260	6675	1317	2722	2670
Reflections unique	1322 (R_int_ = 0.0453)	1187 (R_int_ = 0.0843)	1652 (R_int_ = 0.1731)	1024 (R_int_ = 0.0542)	1511 (R_int_ = 0.0227)	1450 (R_int_ = 0.0327)
Data/restraints/parameters	1322/7/130	1187/7/118	6675/8/1652	1024/10/140	1511/10/140	1450/9/151
Goodness of fit on *F^2^*	1.025	0.972	0.993	1.140	1.085	0.997
Final R_1_ value (*I* > 2σ(*I*))	0.0345	0.0640	0.0849	0.0753	0.0287	0.0414
Final *w*R_2_ value (*I* > 2σ(*I*))	0.0652	0.1192	0.0961	0.1977	0.0706	0.0771
Final R_1_ value (all data)	0.0428	0.1211	0.1835	0.0887	0.0299	0.0537
Final *w*R_2_ value (all data)	0.0675	0.1462	0.1219	0.2292	0.0718	0.0810
CCDC number	2262959	2262961	2262964	2262963	2262960	2262962

**Table 2:** Selected geometric parameters characterizing molecules of compounds **1–5**.

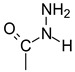				
Compound	d_C=O_ (Å)	d_C-N_ ([Å)	d_N-N_ (Å)	∠ O–C–N–H (°)
**1**	1.232	1.327	1.420	−172.2
**2**	1.220	1.345	1.427	171.5
**3**	1.223	1.325	1.420	−173.5
**4**	1.227	1.336	1.427	165.6
**5**	1.234	1.324	1.414	173.2

**Table 3:** Selected lengths of chemical bonds (Å) in crystals of molecules of compound **6** and L-ascorbic acid (AA) [41].

Compound	d_C1=O_ *	d_C1-C2_	d_C2-O_	d_C2-C3_	d_C3-O_	d_C3-C4_
**6**	1.231	1.409	1.375	1.349	1.287	1.499
AA	1.216	1.452	1.361	1.338	1.326	1.493

* numbering of atoms in accordance with Figure 5.

**Table 4:** Antibacterial activity of the synthesized compounds **1–6**.

Compound	MIC_90_ Values of the Synthesized Compounds 1–6 (µg/mL)			
	Gram-Negative Bacteria		Gram-Positive Bacteria	
	*Escherichia coli* ATCC 25922	*Pseudomonas aeruginosa* ATCC 27853	*Staphylococcus aureus* ATCC 25923	*Staphylococcus aureus* ATCC 29213
**1**	128	128	>512	>512
**2**	512	512	>512	>512
**3**	64	256	>512	>512
**4**	128	256	>512	>512
**5**	128	256	>512	>512
**6**	256	256	512	512

**Table 5:** Antifungal activity of the synthesized compounds **1–6** in different media.

Compound	MIC_90_ Values of the Synthesized Compounds 1–6 (µg/mL)											
	*Candida albicans* SC5314			*Candida glabrata* DSM 11226			*Candida krusei* DSM 6128			*Candida parapsilosis* DSM 5784		
	Medium			Medium			Medium			Medium		
	RPMI + GLC	GPMI − GLC	YNB	RPMI + GLC	GPMI − GLC	YNB	RPMI + GLC	GPMI − GLC	YNB	RPMI + GLC	GPMI − GLC	YNB
**1**	64	64	128	512	256	>512	512	256	>512	512	256	512
**2**	128	64	>512	>512	>512	>512	>512	>512	>512	>512	>512	>512
**3**	32	32	256	512	128	>512	512	128	>512	512	256	512
**4**	128	64	512	>512	256	>512	>512	256	>512	>512	256	512
**5**	128	64	256	>512	512	>512	512	256	>512	512	512	>512
**6**	32	32	128	>512	256	>512	>512	256	>512	>512	256	>512

## Data Availability

Not applicable.

## Supplementary Materials

The following supporting information can be downloaded at: https://www.mdpi.com/article/10.3390/ijms241512114/s1.
