# Supplementary material for: Hydrazinolysis Products of Selected Sugar Lactones—Crystal Structure and Microbiological Activity
Source: Int J Mol Sci. 2023 Jul 28;24(15):12114. doi: 10.3390/ijms241512114 (PMC10418350; doi:10.3390/ijms241512114)
Supplement: Supplementary file 1 [file ijms-24-12114-s001.zip › ijms-2512962-supplementary.pdf]

Supplementary Materials

CONTENTS

Elemental analysis of compounds **1-6**	S3-S4
Hydrogen bonds geometry for compounds **1-6**	S5-S8
Chemical shifts of protons and carbon atoms of derivatives **1-6**	S9-S10
NMR spectra of D-ribonic acid hydrazide (**1**)	S11-S14
NMR spectra of 2-deoxy-D-ribonic acid hydrazide (**2**)	S15-S18
NMR spectra of D-gluconic acid hydrazide (**3**)	S19-S22
NMR spectra of D-fuconic acid hydrazide (**4**)	S23-S26
NMR spectra of L-fuconic acid hydrazide (**5**)	S27-S30
NMR spectra of hydrazinium L-ascorbate (**6**)	S31-S35
ESI MS spectra of **6**	S36-S37
Thermograms (TG) of **6**	S38-S39

Table S1. Results of elemental analysis of compounds **1-6**.

Compound structure	summary formula	molecular weight	N	C	H	N	C	H
			calculated			found		
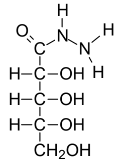	$C_5H_{12}O_5N_2$	180	15.56	33.34	6.67	15.45<br>15.49	33.33<br>33.36	6.627<br>6.633
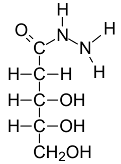	$C_5H_{12}O_4N_2$	164	17.07	36.59	7.32	17.03<br>17.05	36.65<br>36.66	7.275<br>7.240
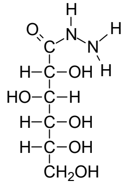	$C_6H_{14}O_6N_2$	210	13.33	34.29	6.67	13.23<br>13.21	34.29<br>34.33	6.625<br>6.632
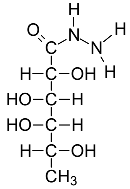	$C_6H_{14}O_5N_2$	194	14.43	37.11	7.22	14.33<br>14.38	37.02<br>37.01	7.236<br>7.290

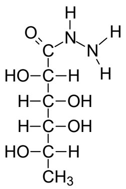	$C_6H_{14}O_5N_2$	194	14.43	37.11	7.22	14.37<br>14.39	37.07<br>37.08	7.238<br>7.280
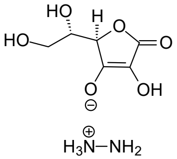	$C_6H_{12}O_6N_2$	208	13.46	34.62	5.77	13.43<br>13.48	34.65<br>34.69	5.739<br>5.754

Table S2. Hydrogen bonds geometry for compounds **1-3**.

	**D–H···A**	***d*(D–H) [Å]**	***d*(H···A) [Å]**	***d*(D···A) [Å]**	**∠D–H···A (°)**
**1**	N1–H1A···O2^{i}	0.88(3)	2.45(3)	3.227(3)	147(2)
	N1–H1B···O3^{ii}	0.87(3)	2.49(3)	3.331(3)	165(2)
	O2–H2A···O4^{iii}	0.80(3)	1.93(3)	2.694(3)	160(4)
	N2–H2B···O2*	0.86(3)	2.18(3)	2.576(3)	108(2)
	N2–H2B···O3^{iv}	0.86(3)	2.11(3)	2.931(3)	158(3)
	O3–H3A···O5^{ii}	0.83(3)	2.08(3)	2.738(3)	136(3)
	O4–H4A···O1^{v}	0.81(2)	1.85(2)	2.658(3)	174(4)
	O5–H5A···N1^{vi}	0.82(3)	2.09(3)	2.887(3)	165(4)
	C3–H3B···O1*	0.98	2.60	2.979(3)	103
	C4–H4B···O2*	0.98	2.54	2.902(3)	101
	C5–H5C···O3*	0.97	2.54	2.879(3)	100
Symmetry code: (i) 1+x,y,z; (ii) 1/2-x,-y,1/2+z; (iii) -1+x,y,z; (iv) -1-x,-1/2+y,-3/2-z; (v) -x,-1/2+y,-3/2-z; (vi) -x,1/2+y,-3/2-z.					
**2**	N1–H1A···O3^{i}	0.87(7)	2.68(7)	3.289(9)	128(6)
	N1–H1B···O5^{ii}	0.85(6)	2.24(5)	3.086(9)	169(8)
	N2–H2A···O1^{iii}	0.88(4)	2.00(4)	2.843(8)	159(8)
	O3–H3A···N1^{i}	0.82(8)	2.01(8)	2.806(9)	164(8)
	O4–H4A···O3^{iv}	0.81(7)	2.10(7)	2.887(9)	162(7)
	O5–H5A···O5^{v}	0.82(5)	1.93(6)	2.720(8)	163(9)
	C3–H3B···O4^{vi}	0.98	2.51	3.315(9)	139
	C5–H5C···O4^{vi}	0.97	2.54	3.312(9)	136
Symmetry code: (i) -x,1/2+y,1-z; (ii) x,y,1+z; (iii) x,-1+y,z; (iv) x,1+y,z; (v) -x,-1/2+y,-z; (vi) 1-x,-1/2+y,-z.					
**3**	N1–H1A···O2^{i}	0.86(6)	2.12(7)	2.904(9)	150(7)
	N1–H1B···O5^{ii}	0.87(6)	2.34(6)	3.134(9)	153(8)

O2–H2A···O4*	0.82(6)	1.86(6)	2.655(8)	164(7)
N2–H2B···O2*	0.88(5)	2.17(6)	2.518(8)	103(5)
N2–H2B···O5^{iii}	0.82(5)	2.05(5)	2.885(9)	157(5)
O3–H3A···O4^{i}	0.82(4)	1.98(4)	2.763(7)	160(7)
O4–H4A···O1^{iii}	0.82(6)	1.83(5)	2.608(8)	156(8)
O5–H5A···O6^{i}	0.82(4)	1.93(4)	2.697(7)	157(8)
O6–H6A···N1^{iv}	0.82(7)	2.01(6)	2.796(9)	160(9)
C2–H2C···O3^{iv}	0.98	2.49	3.322(9)	142
C5–H5B···O3*	0.98	2.55	2.890(9)	100

Symmetry code: (i) 1+x,y,z; (ii) 1/2-x,2-y,1/2+z; (iii) -x,-1/2+y,1/2-z; (iv) -x,1/2+y,1/2-z.

Table S3. Hydrogen bonds geometry for compounds **4-6**.

	**D–H···A**	***d*(D–H) [Å]**	***d*(H···A) [Å]**	***d*(D···A) [Å]**	**∠D–H···A (°)**
**4**	N1–H1A···O5^{i}	0.86(6)	2.62(9)	3.139(9)	120(9)
	N1–H1B···O2^{ii}	0.87(7)	2.38(7)	3.225(9)	168(9)
	O2–H2A···O3^{iii}	0.84(9)	1.94(9)	2.770(8)	168(9)
	N2–H2B···O2*	0.86(8)	2.18(9)	2.633(9)	113(8)
	N2–H2B···O5^{iv}	0.86(8)	2.44(9)	3.054(9)	129(8)
	O3–H3A···O1^{v}	0.83(9)	1.94(8)	2.714(9)	155(9)
	O4–H4A···O5*	0.83(9)	2.49(9)	2.824(9)	105(6)
	O4–H4A···N1^{vi}	0.83(9)	2.25(9)	2.902(9)	135(9)

	O5–H5A···O4^{vii}	0.84(9)	1.97(9)	2.744(9)	153(9)
	C2–H2C···O1^{iii}	0.98	2.58	3.320(9)	132
	C2–H2C···O4*	0.98	2.45	2.874(9)	105
	C3–H3B···O1*	0.98	2.44	2.805(9)	101
	C4–H4B···O1^{v}	0.98	2.52	3.176(9)	124
Symmetry code: (i) x,y,1+z; (ii) x,1+y,z; (iii) -1+x,y,z; (iv) -1+x,y,1+z; (v) x,-1+y,z; (vi) x,y,-1+z; (vii) 1+x,y,z.					
**5**	N1–H1A···O2^{i}	0.86(3)	2.40(3)	3.230(3)	163(3)
	N1–H1B···O5^{ii}	0.86(3)	2.56(3)	3.120(4)	124(3)
	O2–H2A···O3^{iii}	0.81(4)	1.95(4)	2.755(3)	170(4)
	N2–H2B···O2*	0.84(3)	2.29(3)	2.629(3)	105(3)
	N2–H2B···O5^{iv}	0.84(3)	2.29(3)	3.039(3)	149(3)
	O3–H3A···O1^{v}	0.86(3)	1.89(3)	2.717(2)	161(3)
	O4–H4A···O5*	0.80(3)	2.45(3)	2.822(3)	110(3)
	O4–H4A···N1^{vi}	0.80(3)	2.14(3)	2.890(3)	157(3)
	O5–H5A···O4^{vii}	0.82(4)	1.92(4)	2.734(3)	177(3)
	C2–H2C···O1^{iii}	0.98	2.57	3.298(3)	131
	C2–H2C···O4*	0.98	2.43	2.856(3)	105
	C3–H3B···O1*	0.98	2.43	2.793(3)	101
	C4–H4B···O1^{v}	0.98	2.53	3.182(3)	124
	C5–H5B···O3*	0.98	2.57	2.950(4)	103
Symmetry code: (i) x,-1+y,z; (ii) x,y,-1+z; (iii) 1+x,y,z; (iv) 1+x,y,-1+z; (v) x,1+y,z; (vi) x,y,1+z; (vii) -1+x,y,z.					
**6**	N1–H1A···O2^{i}	0.88(4)	2.45(3)	3.227(3)	147(2)
	N1–H1B···O5^{ii}	0.86(4)	2.49(3)	3.331(3)	165(2)

N1–H1B···O6^{ii}	0.87(3)	2.49(3)	3.331(3)	165(2)
N2–H2A···O1	0.86(3)	2.18(3)	2.576(3)	108(2)
N2–H2B···N1^{iii}	0.86(3)	2.11(3)	2.931(3)	158(3)
N2–H2C···O3^{iv}	0.86(3)	2.11(3)	2.931(3)	158(3)
N2–H2C···O5^{iv}	0.86(3)	2.11(3)	2.931(3)	158(3)
O2–H2D···O3^{iv}	0.80(3)	1.93(3)	2.694(3)	160(4)
O5–H5A···O3^{i}	0.82(3)	2.09(3)	2.887(3)	165(4)
O6–H6A···O6^{v}	0.82(3)	2.09(3)	2.887(3)	165(4)
C4–H4A···O4^{vi}	0.98	2.54	2.902(3)	101

Symmetry code: (i) x,-1+y,z; (ii) 1+x,y,z; (iii) 2-x,1/2+y,1-z; (iv) 1-x,-1/2+y,1-z; (v) -x,1/2+y,2-z; (vi) 1-x,1/2+y,2-z.

Table S4. Chemical shifts of protons and carbon atoms of derivatives **1-6 (**numbering of atoms in accordance with **Figure 4**).

Compound structure	H2	H3	H4	H5	H6	C1	C2	C3	C4	C5	C6
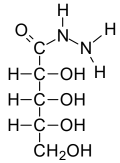	*d*<br>4.32	*dd*<br>3.67	*m*<br>3.79-3.76	*dd*<br>3.74	-	172.51	72.13	72.66	70.94	62.83	-
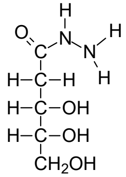	*dd*<br>3.69	*m*<br>3.56	*m*<br>3.96	*dd*<br>2.51<br>*dd*<br>2.28	-	172.92	37.47	74.26	68.77	62.38	-
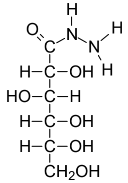	*dd*<br>4.30	*m*<br>4.05-4.01	*m*<br>4.67-4.63	*m*<br>3.72-3.67	*m*<br>3.79-3-74<br>*m*<br>3.62-3.57	172.65	72.85	70.39	71.74	71.05	62.63
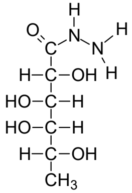	*s*<br>4.45	*d*<br>3.91	*d*<br>3.43	*d*<br>4.05	*d*<br>1.20	173.91	70.86	71.11	72.51	65.82	18.69
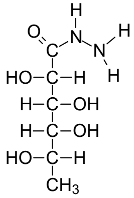	*s*<br>4.45	*d*<br>3.91	*d*<br>3.43	*d*<br>4.05	*d*<br>1.20	173.91	70.86	71.11	72.51	65.82	18.69
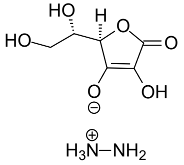	-	-	*d*<br>4.42	*m*<br>3.92	*m*<br>3.65	177.51	113.09	175.59	78.35	69.52	62.52

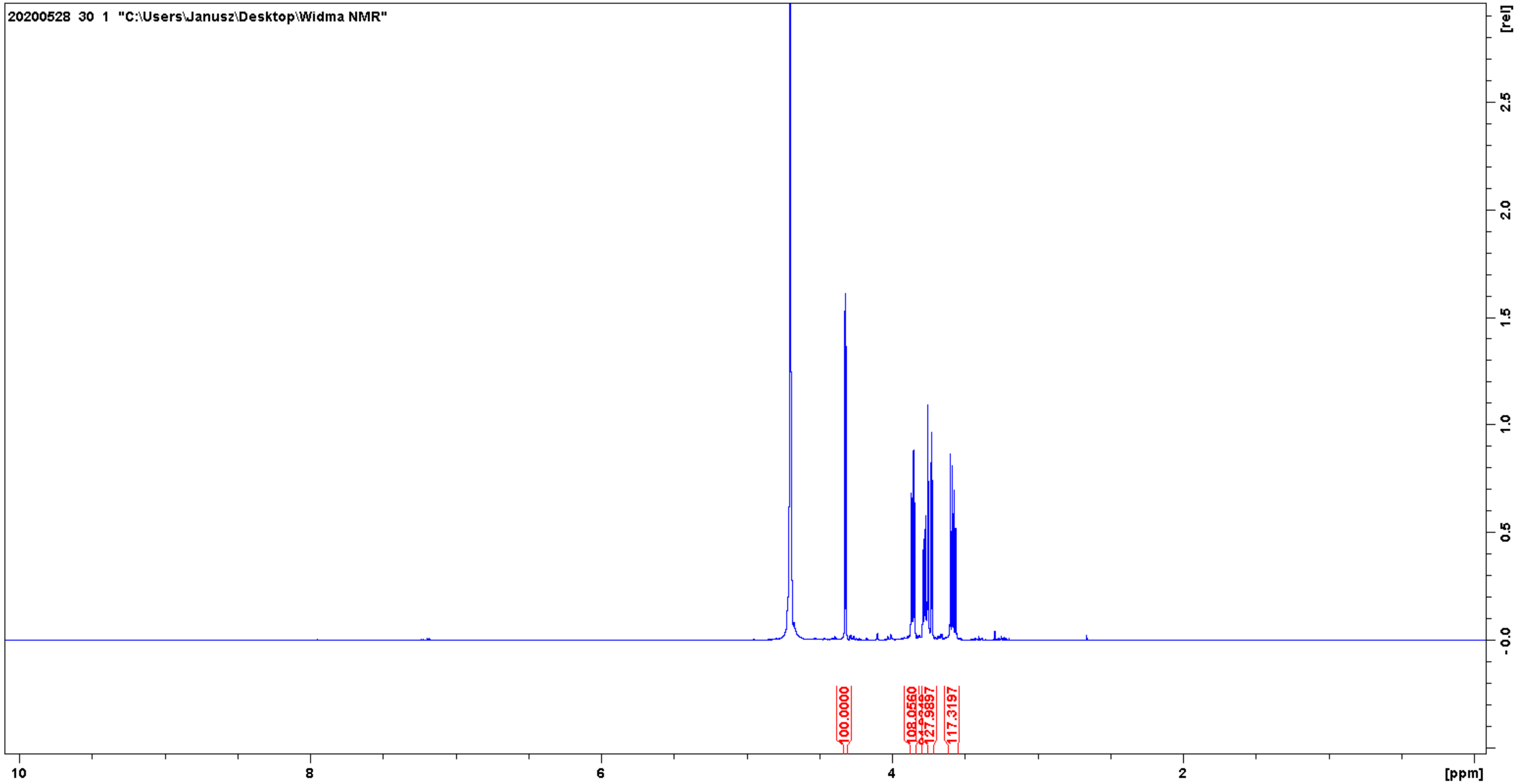

Figure S1. ^{1}H NMR spectrum (D_2O, 500 MHz) of D-ribonic acid hydrazide (**1**).

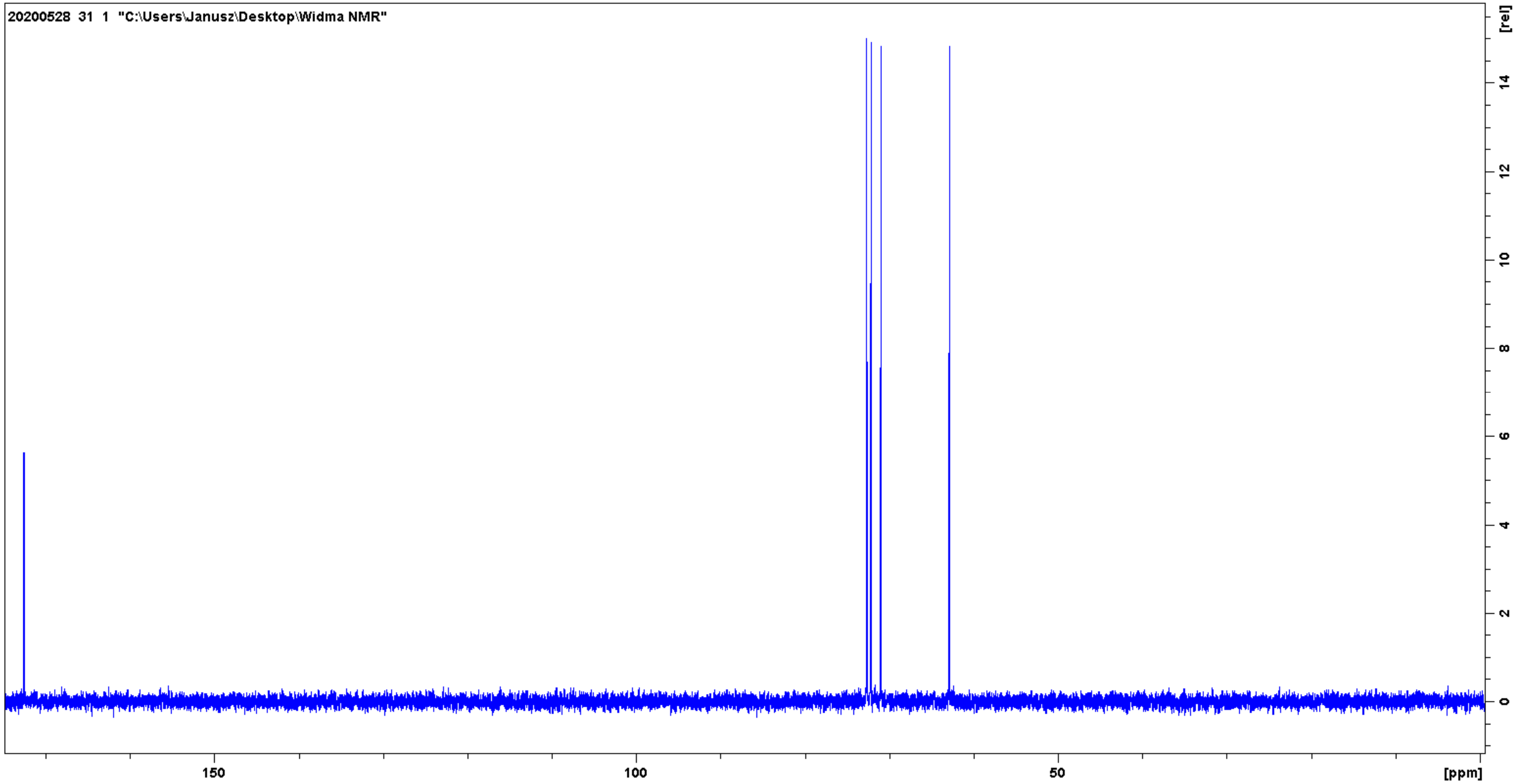

Figure S2. ^{13}C NMR spectrum (D_2O, 125 MHz) of D-ribonic acid hydrazide (**1**).

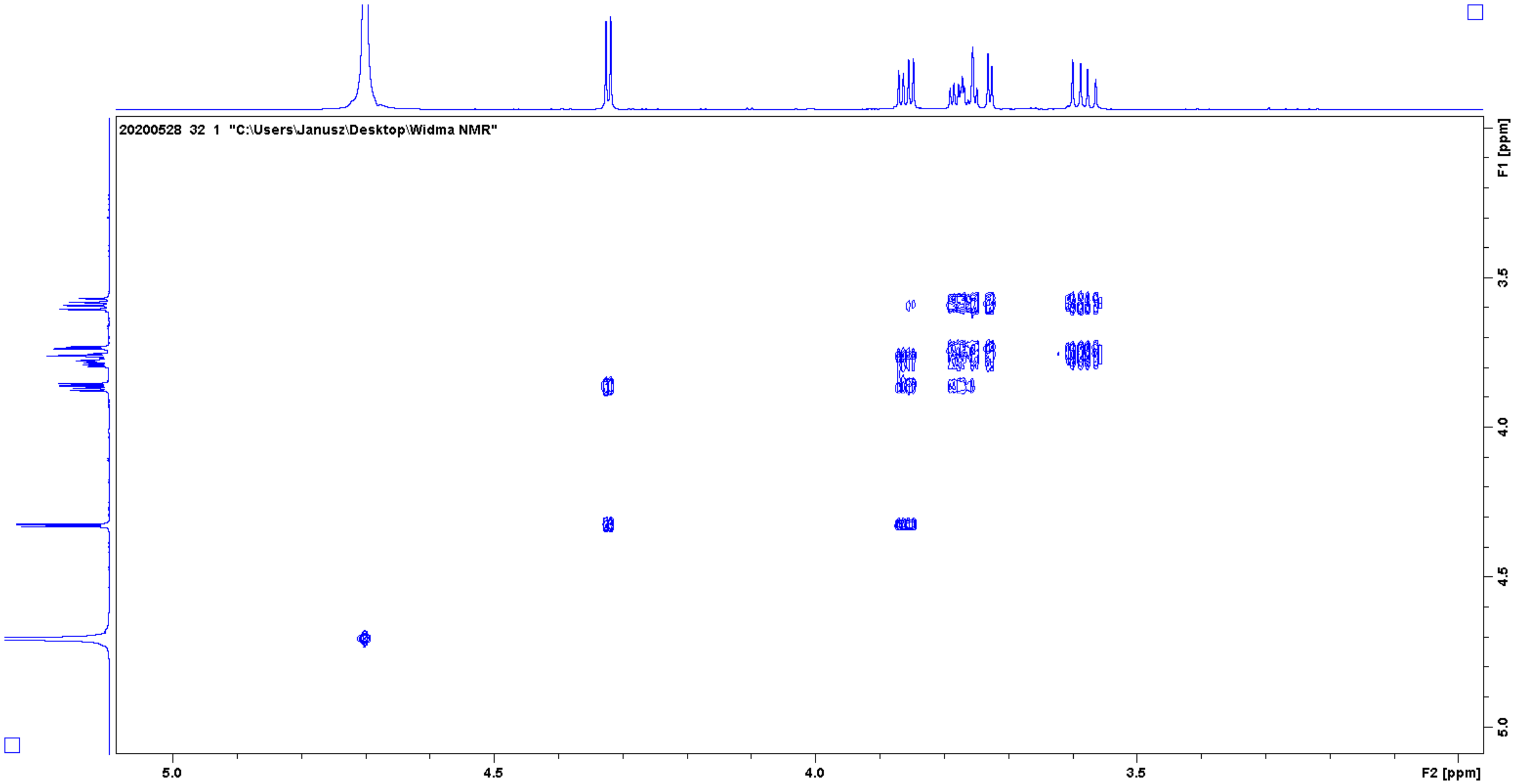

Figure S3. COSY spectrum of D-ribonic acid hydrazide (**1**).

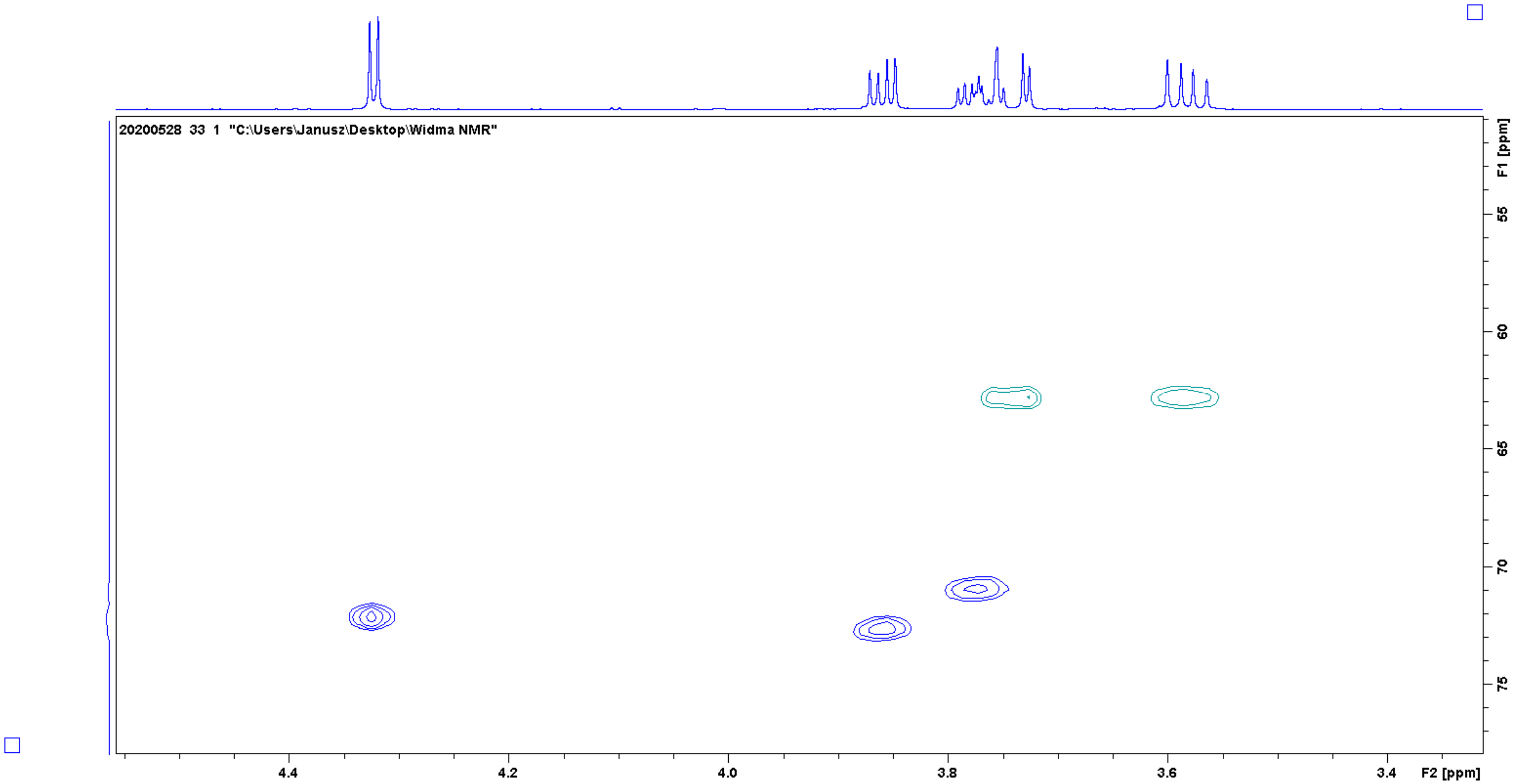

Figure 4S. HSQC DEPT spectrum of D-ribonic acid hydrazide (**1**).

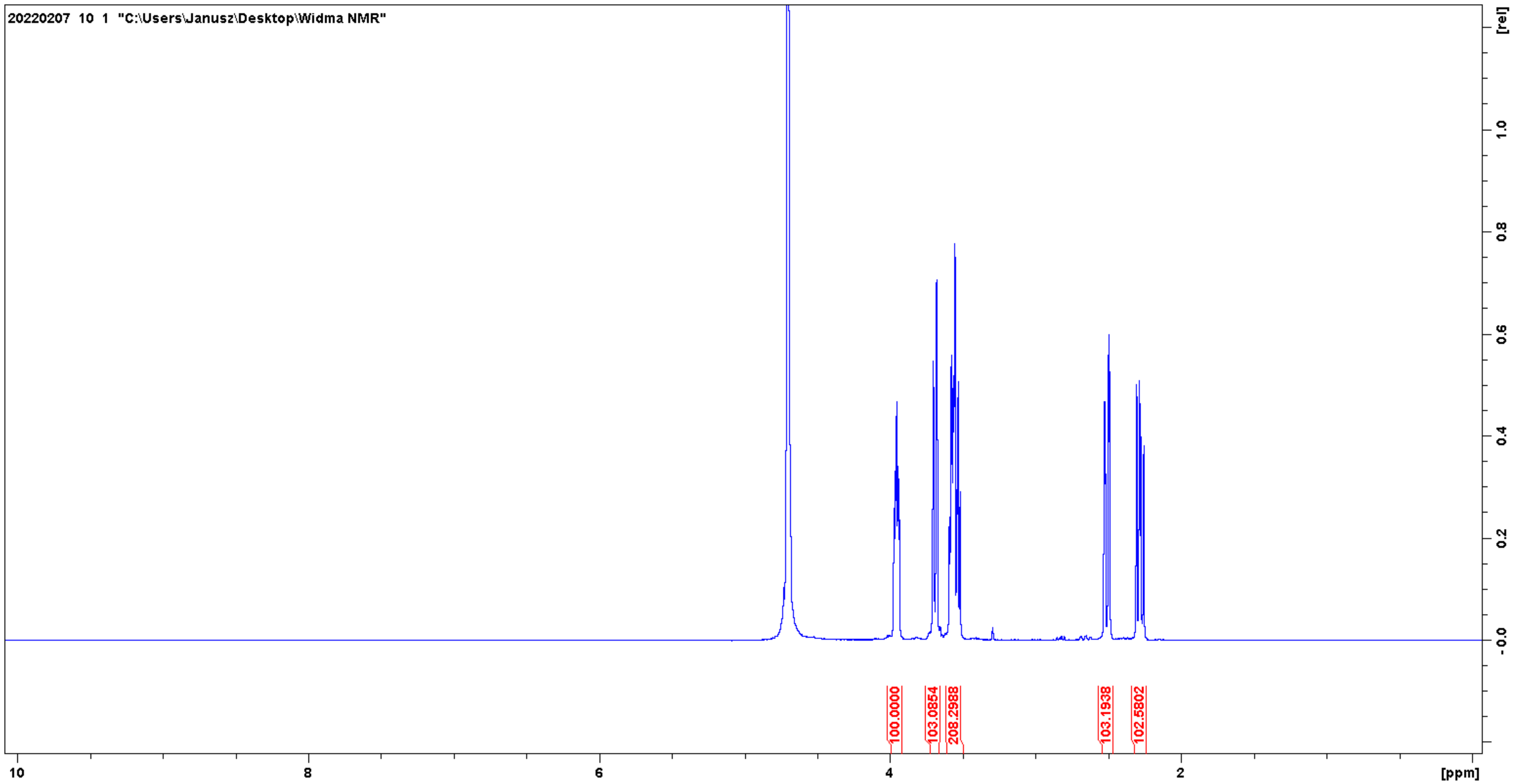

Figure S5. ^{1}H NMR spectrum (D_2O, 500 MHz) of 2-deoxy-D-ribonic acid hydrazide (**2**).

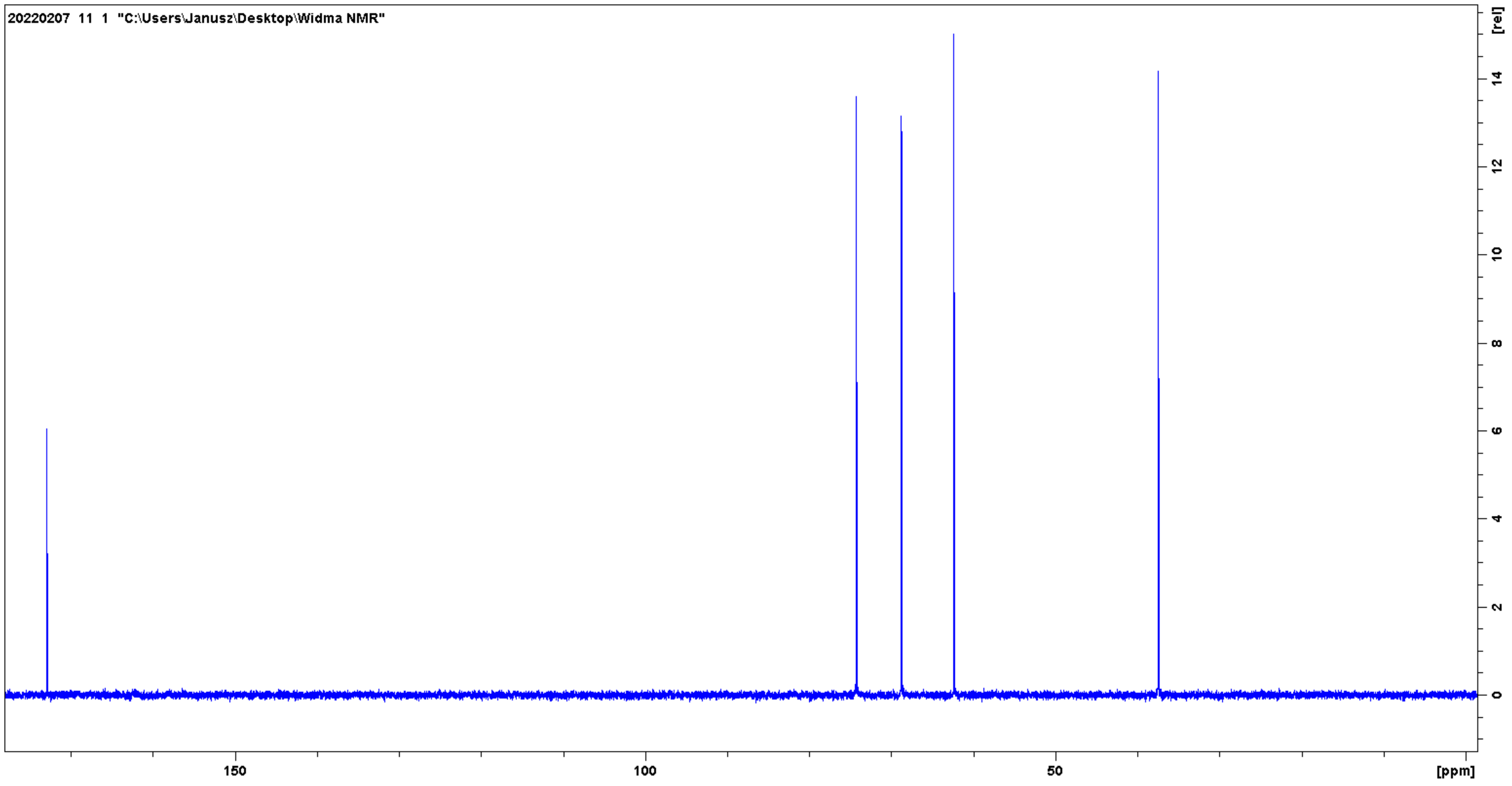

Figure S6. ^{13}C NMR spectrum (D_2O, 125 MHz) of 2-deoxy-D-ribonic acid hydrazide (**2**).

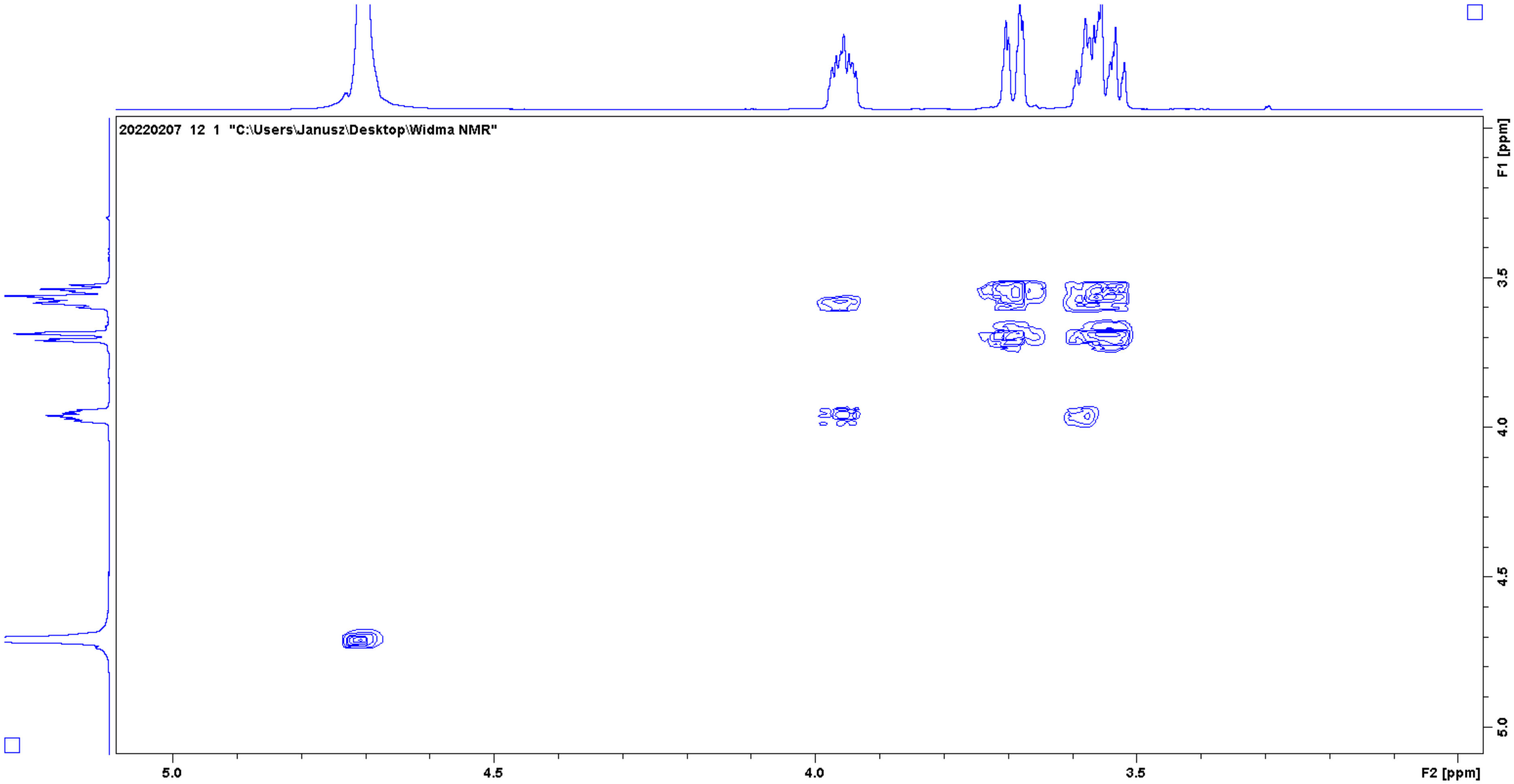

Figure S7. COSY spectrum of 2-deoxy-D-ribonic acid hydrazide (**2**).

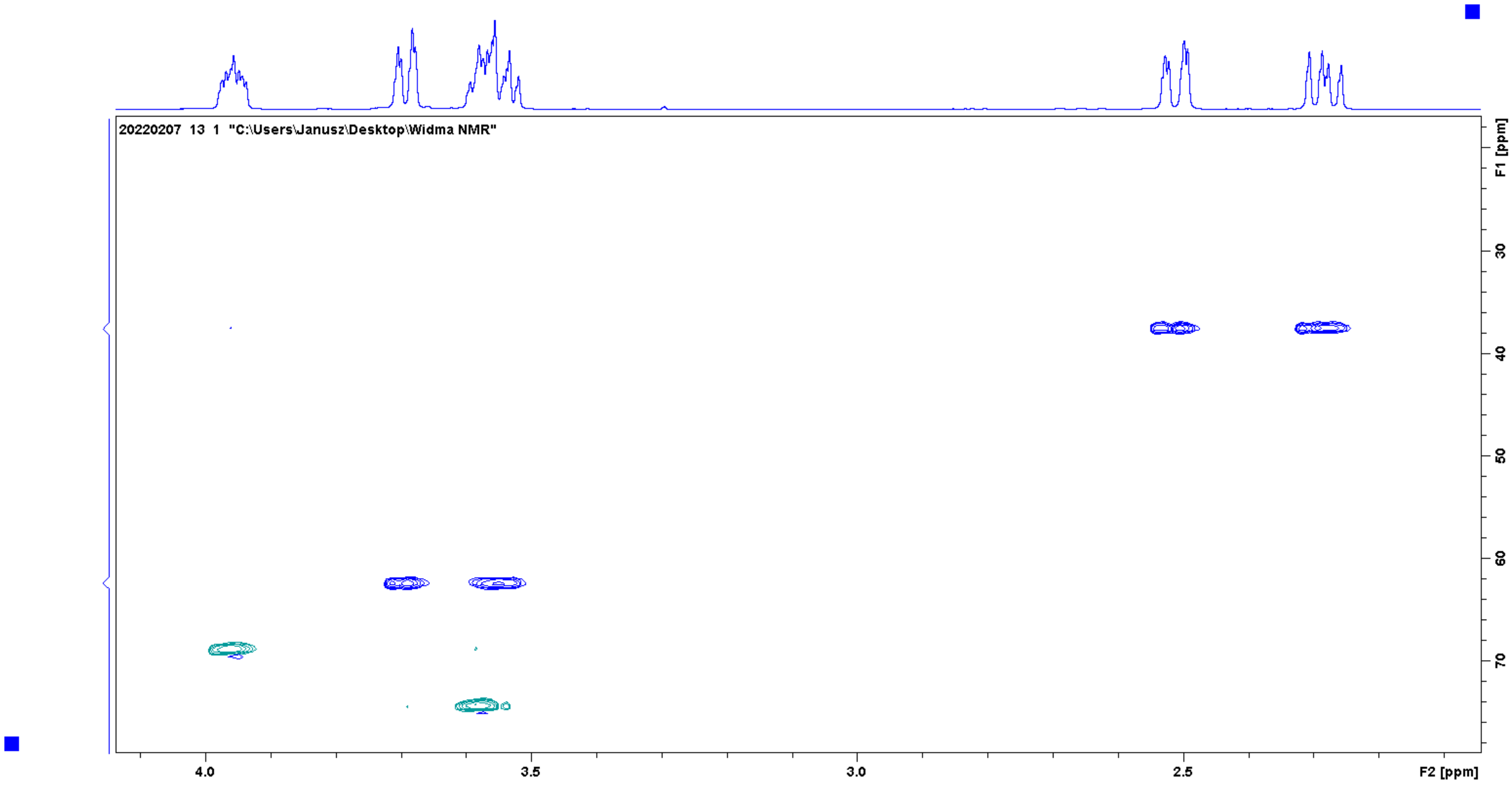

Figure S8. HSQC DEPT spectrum of 2-deoxy-D-ribonic acid hydrazide (**2**).

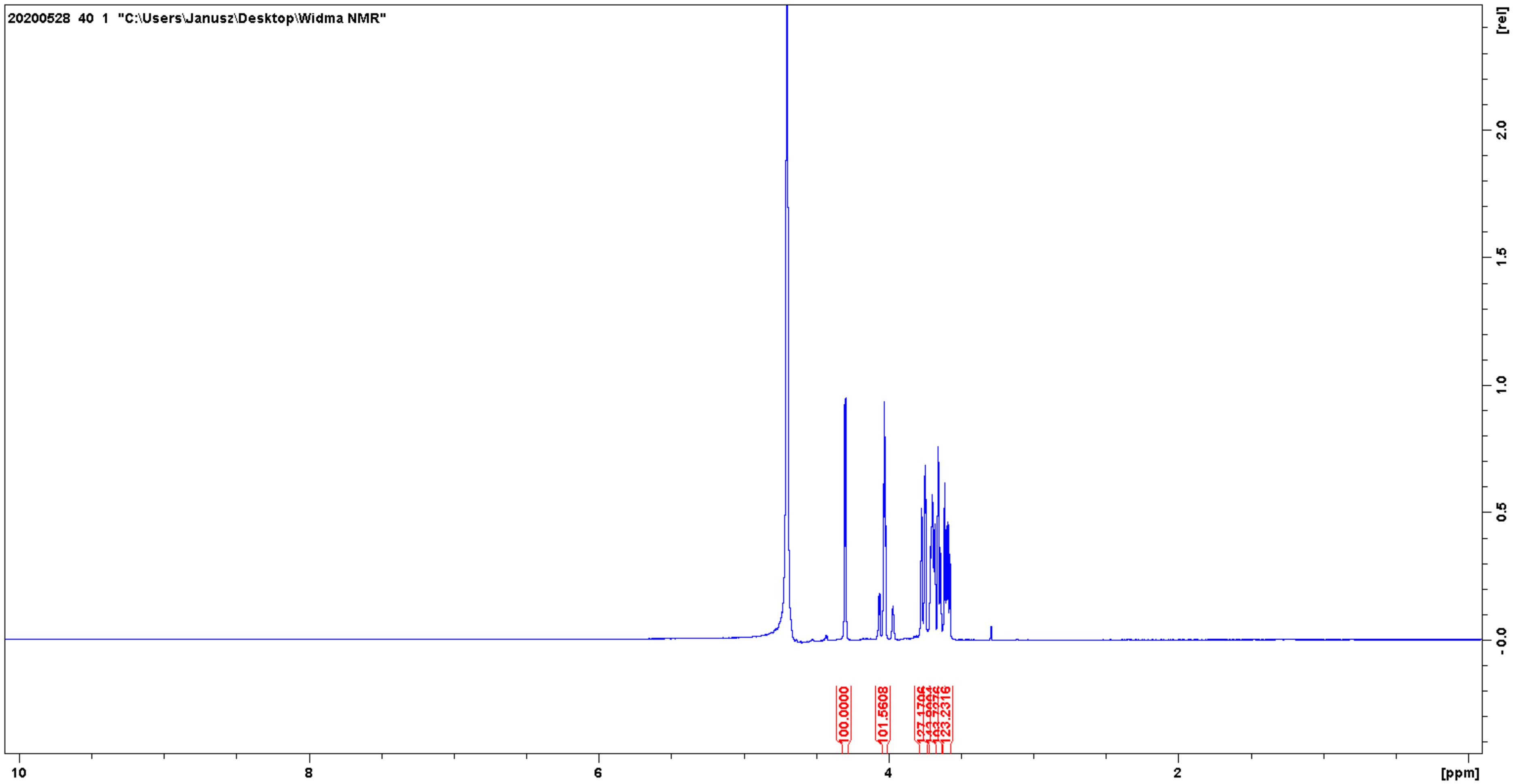

Figure S9. ^{1}H NMR spectrum (D_2O, 500 MHz) of D-gluconic acid hydrazide (**3**).

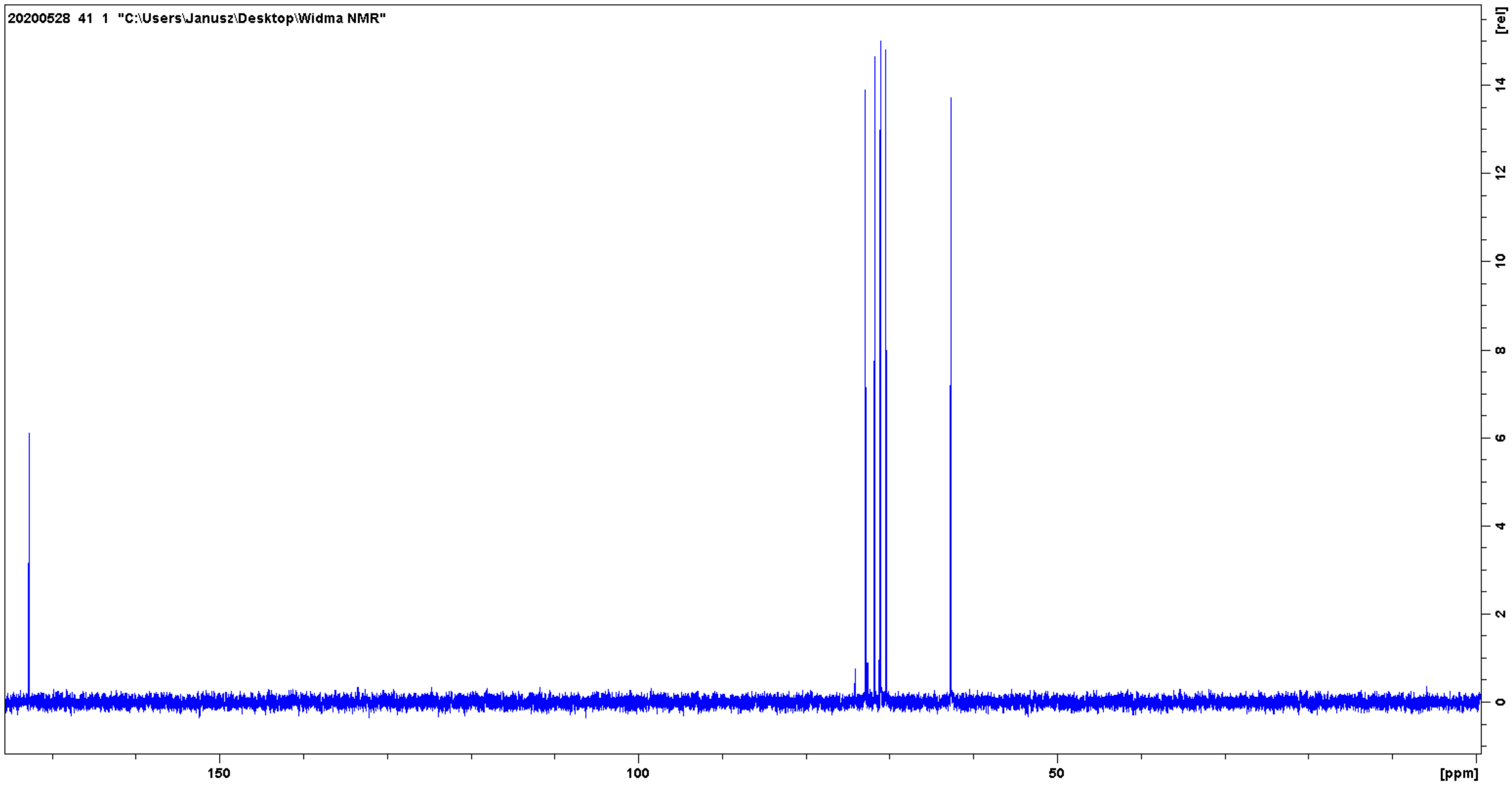

Figure S10. ^{13}C NMR spectrum (D_2O, 125 MHz) of D-gluconic acid hydrazide (**3**).

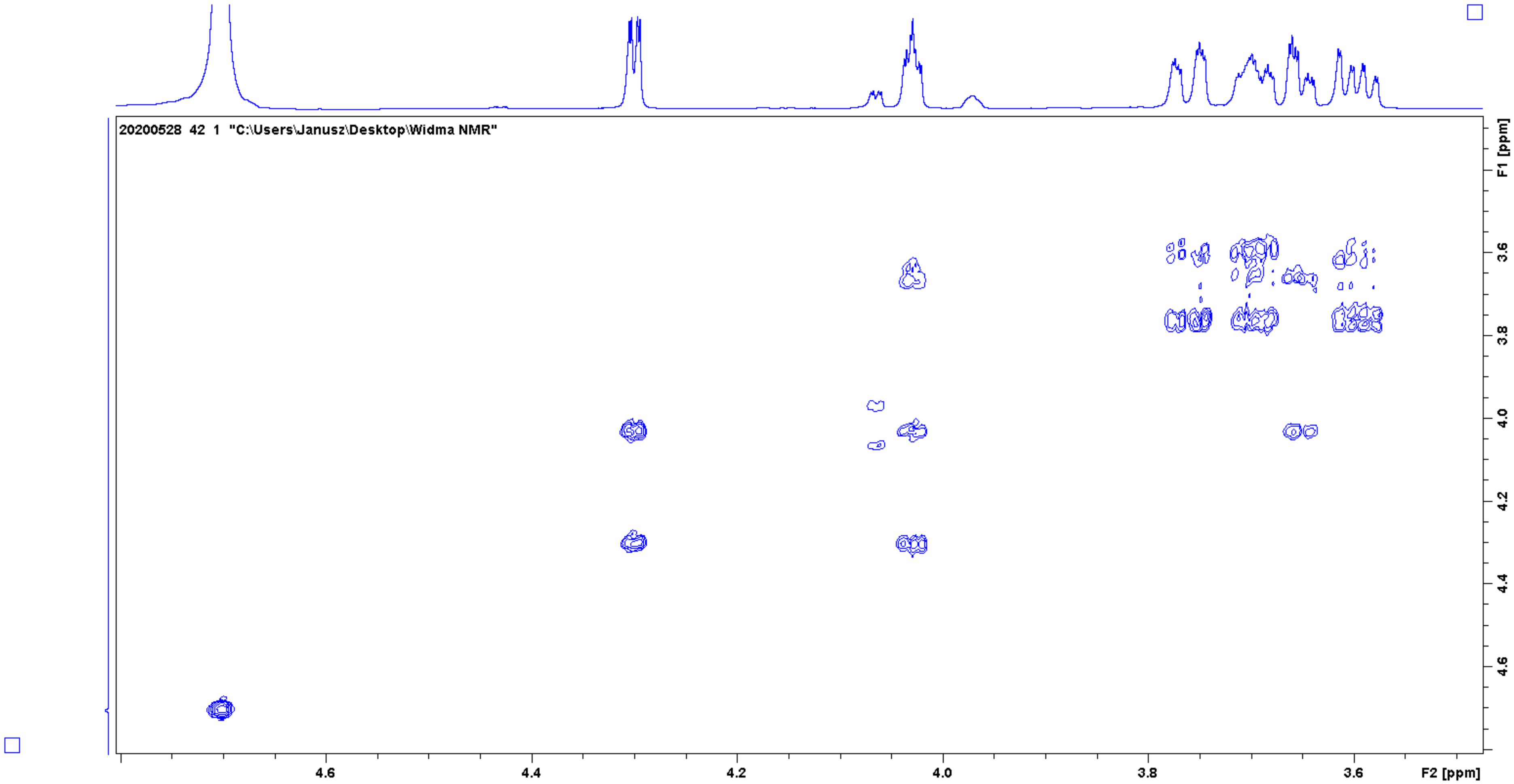

Figure S11. COSY spectrum of D-gluconic acid hydrazide (**3**).

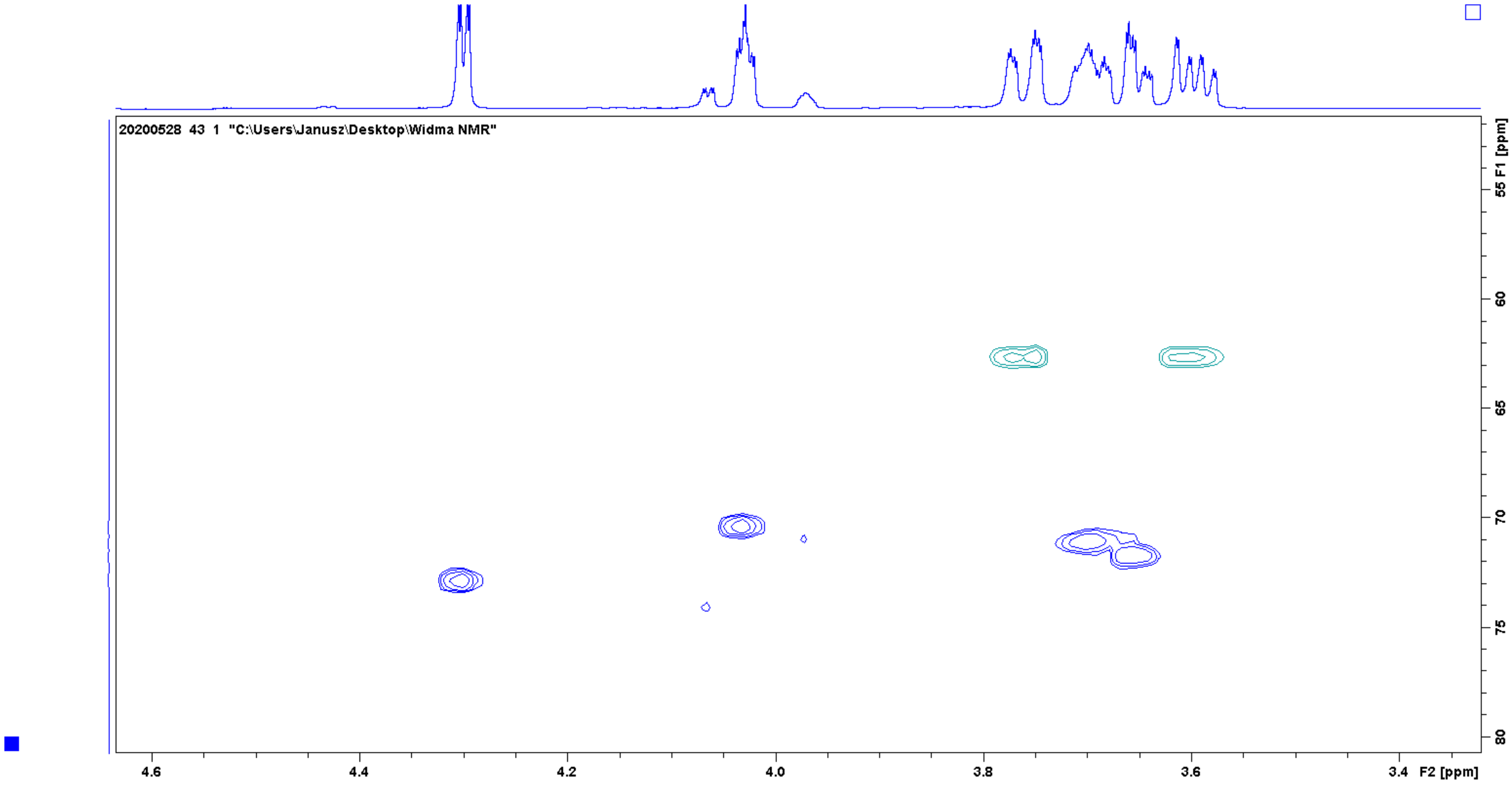

Figure S12. HSQC DEPT spectrum of D-gluconic acid hydrazide (**3**).

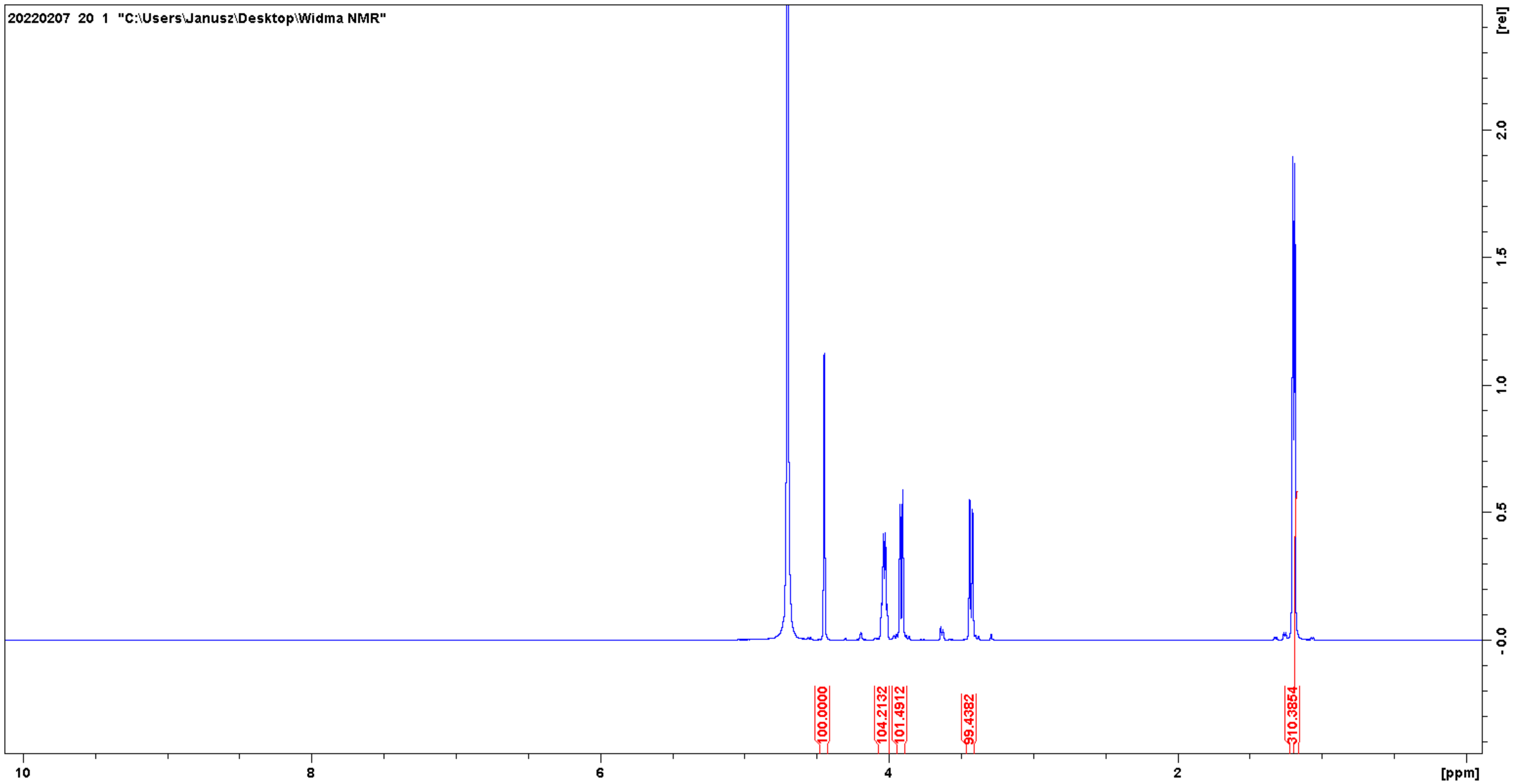

Figure S13. ^{1}H NMR spectrum (D_2O, 500 MHz) of D-fuconic acid hydrazide (**4**).

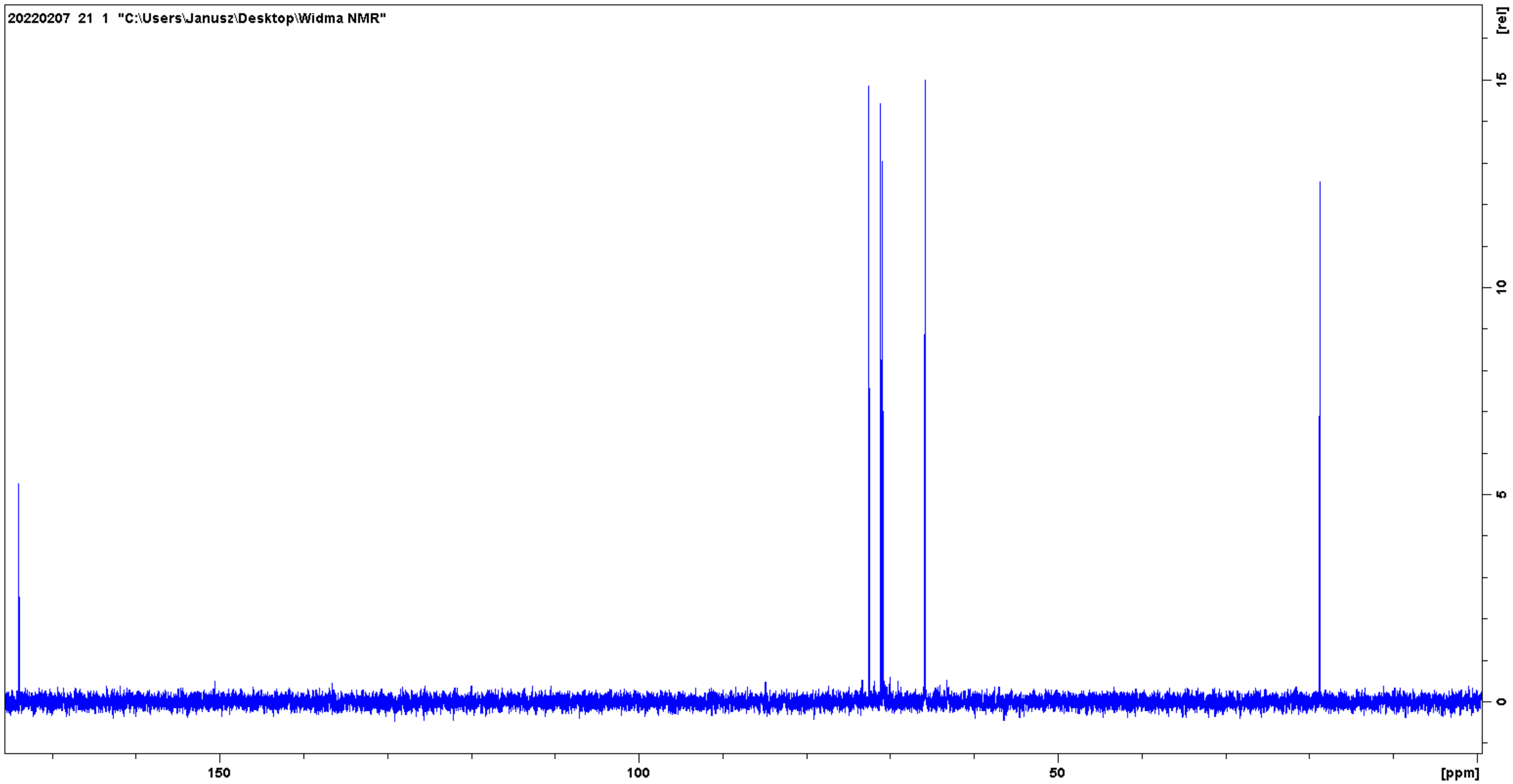

Figure S14. ^{13}C NMR spectrum (D_2O, 125 MHz) of D-fuconic acid hydrazide (**4**).

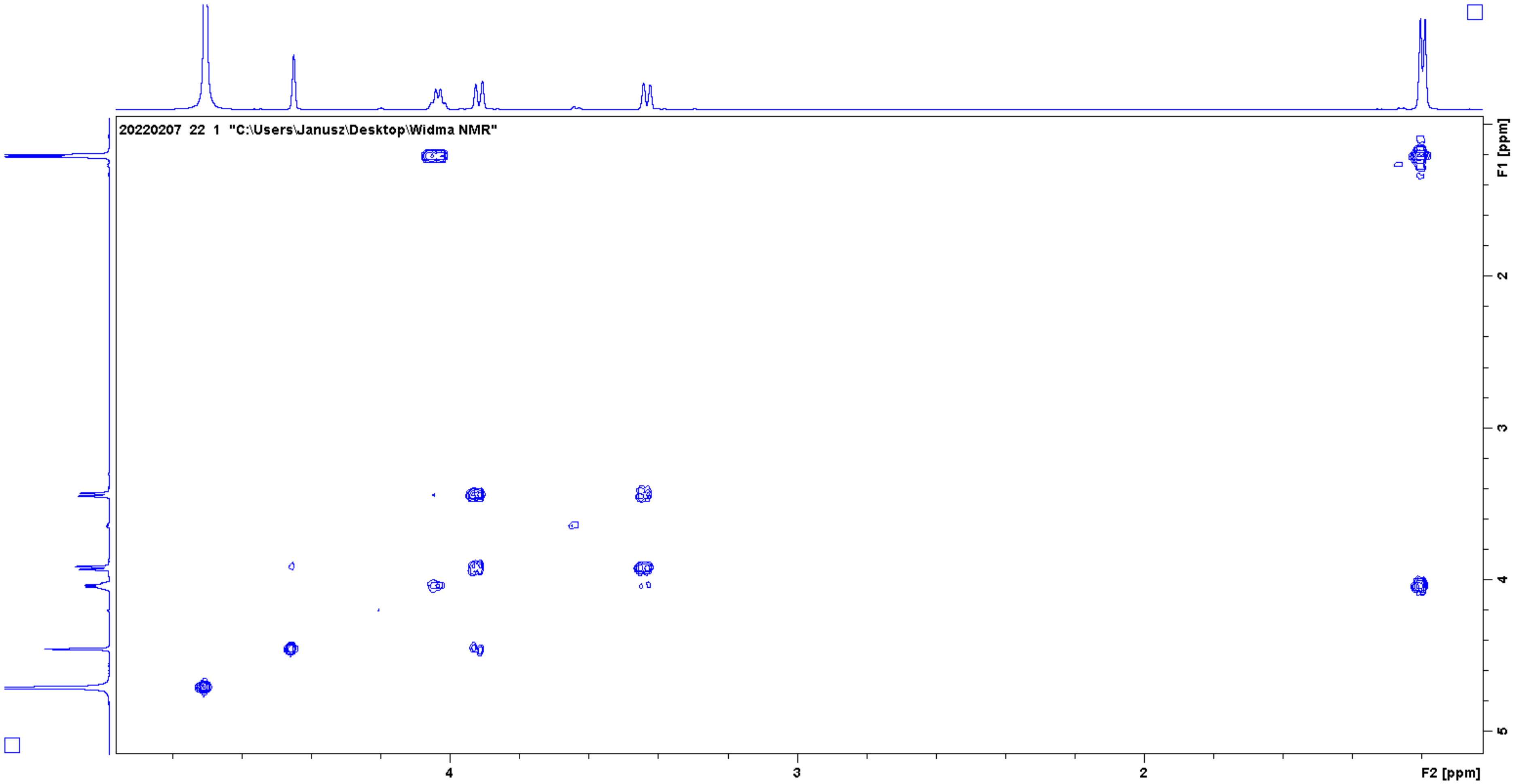

Figure S15. COSY spectrum of D-fuconic acid hydrazide (**4**).

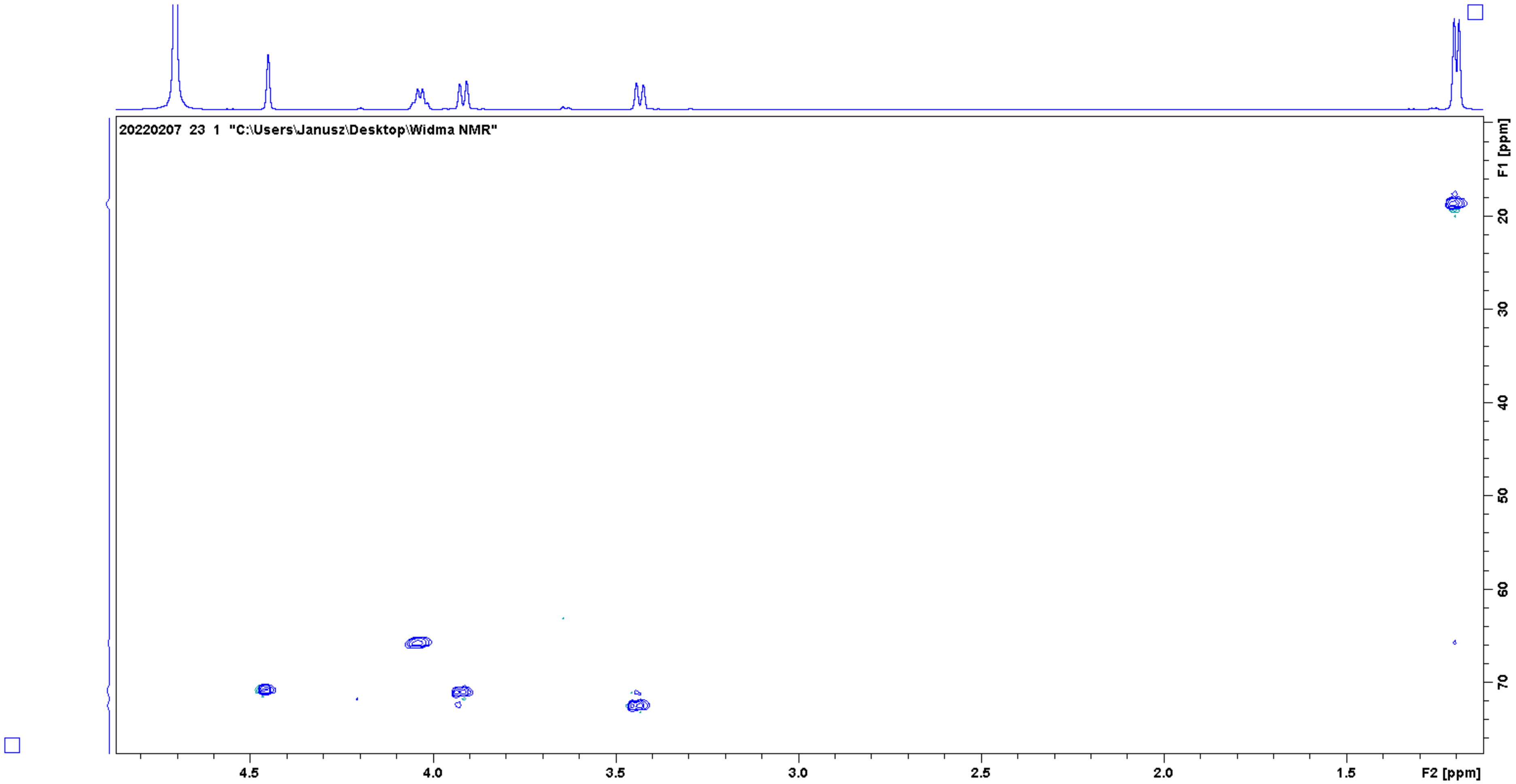

Figure S16. HSQC DEPT spectrum of D-fuconic acid hydrazide (**4**).

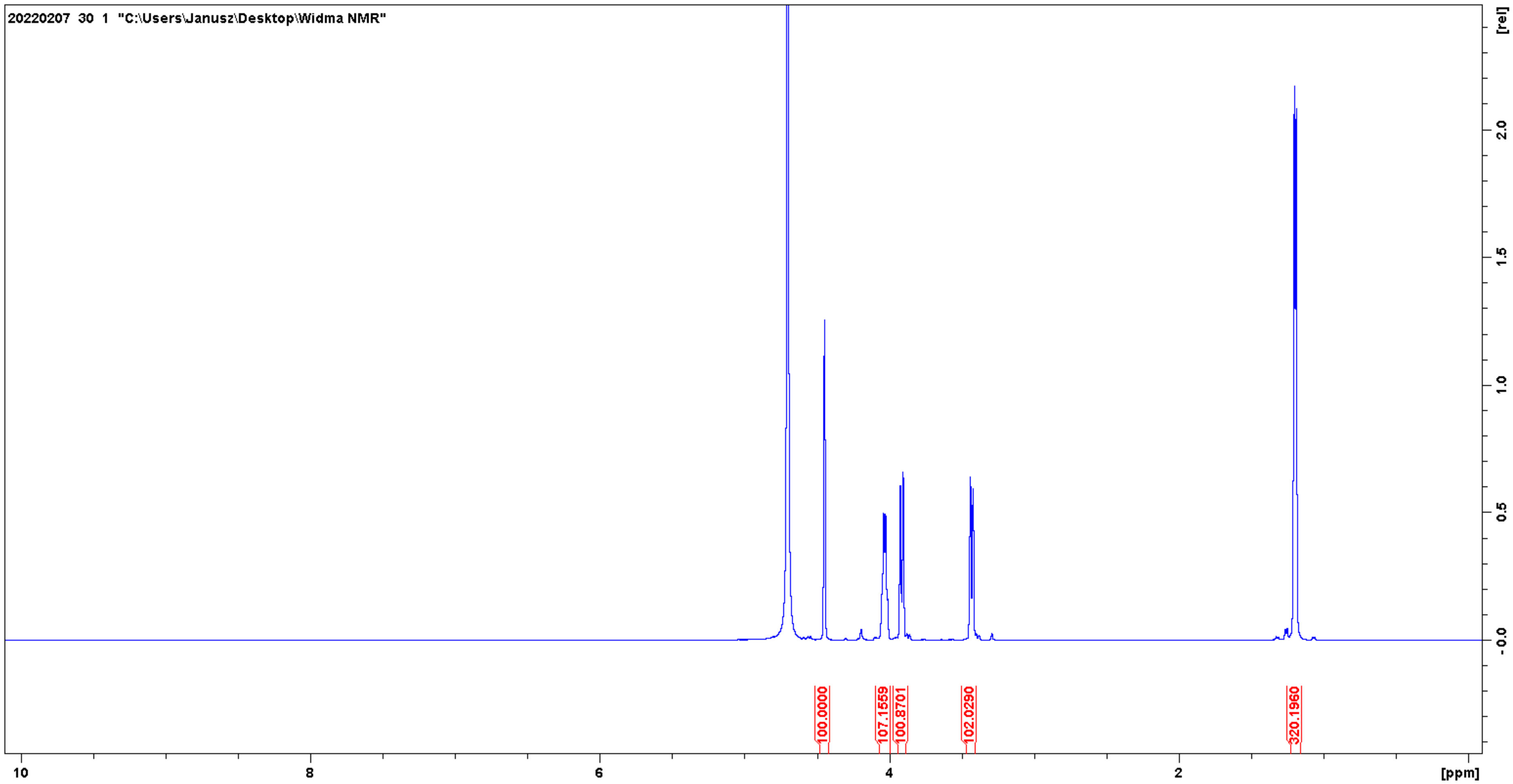

Figure S17. ^{1}H NMR spectrum (D_2O, 500 MHz) of L-fuconic acid hydrazide (**5**).

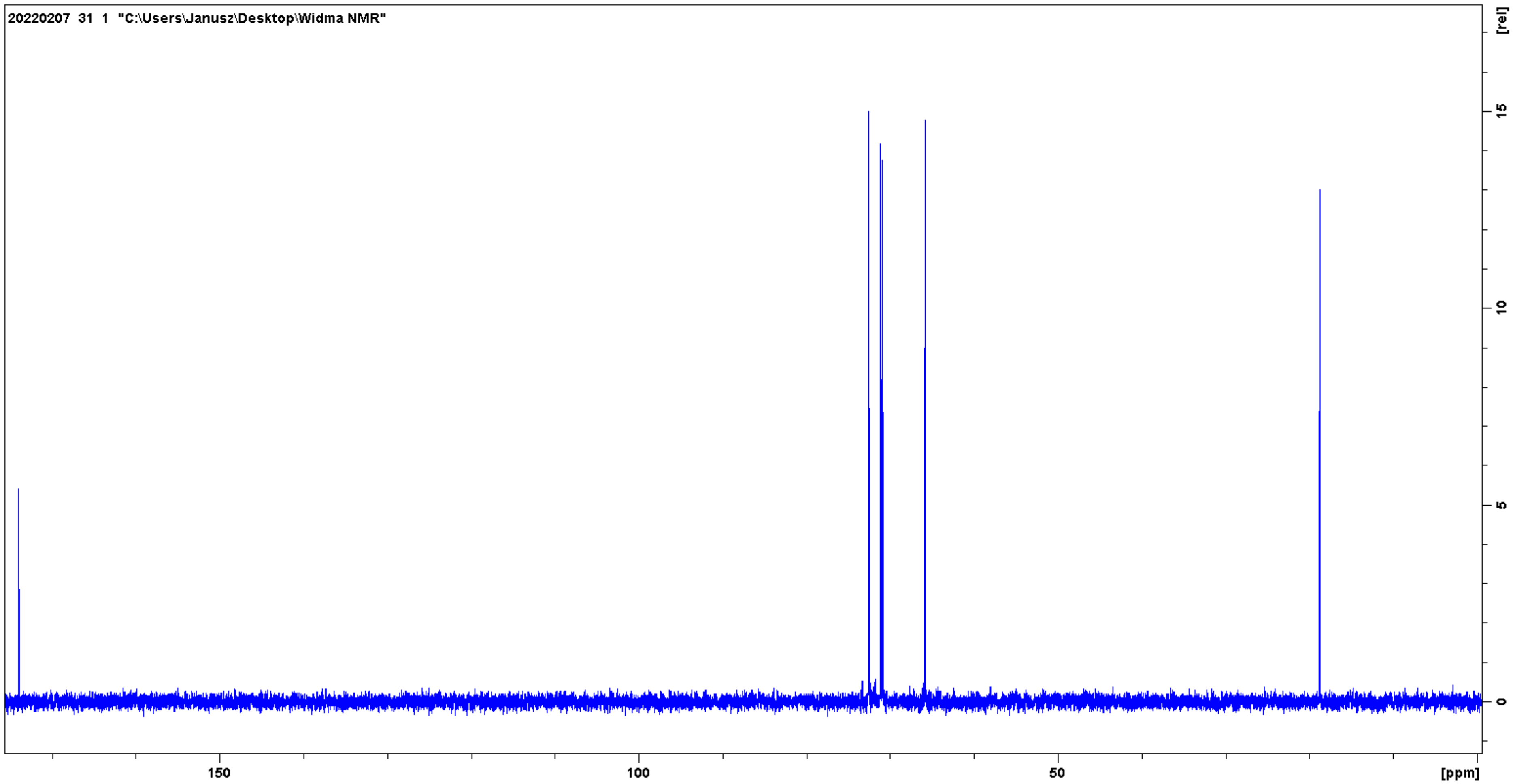

Figure S18. ^{13}C NMR spectrum (D_2O, 125 MHz) of L-fuconic acid hydrazide (**5**).

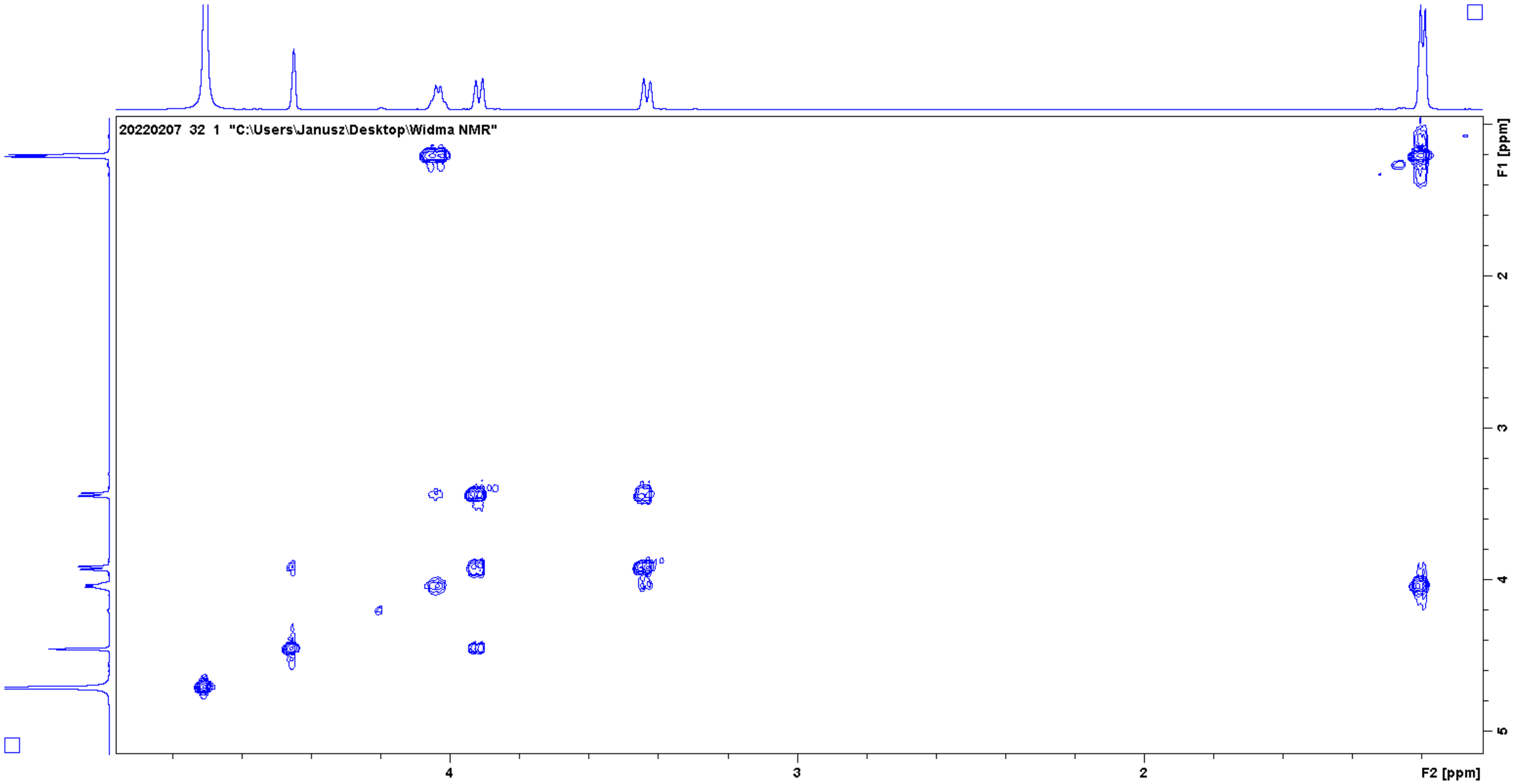

Figure S19. COSY spectrum of L-fuconic acid hydrazide (**5**).

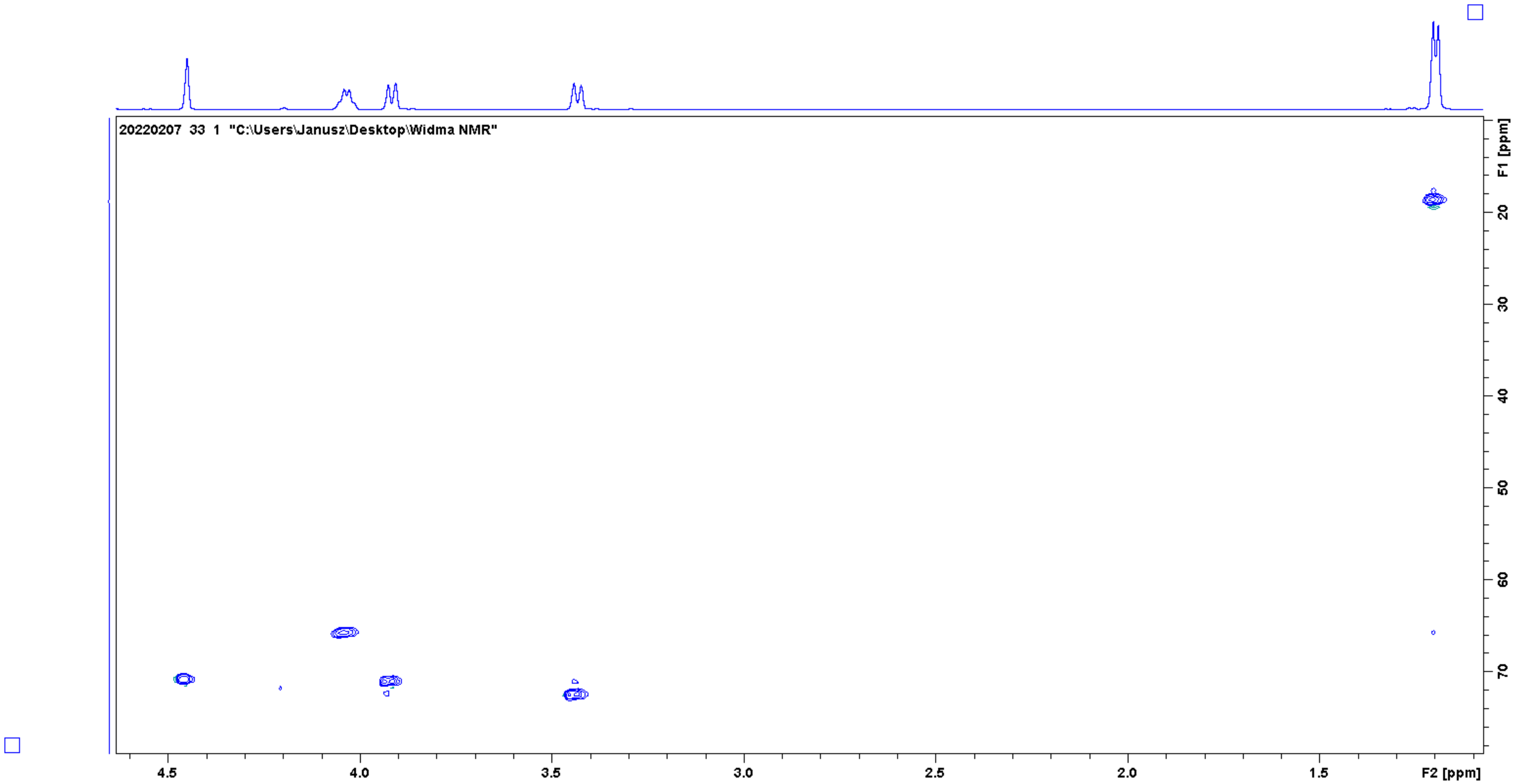

Figure S20. HSQC DEPT spectrum of L-fuconic acid hydrazide (**5**).

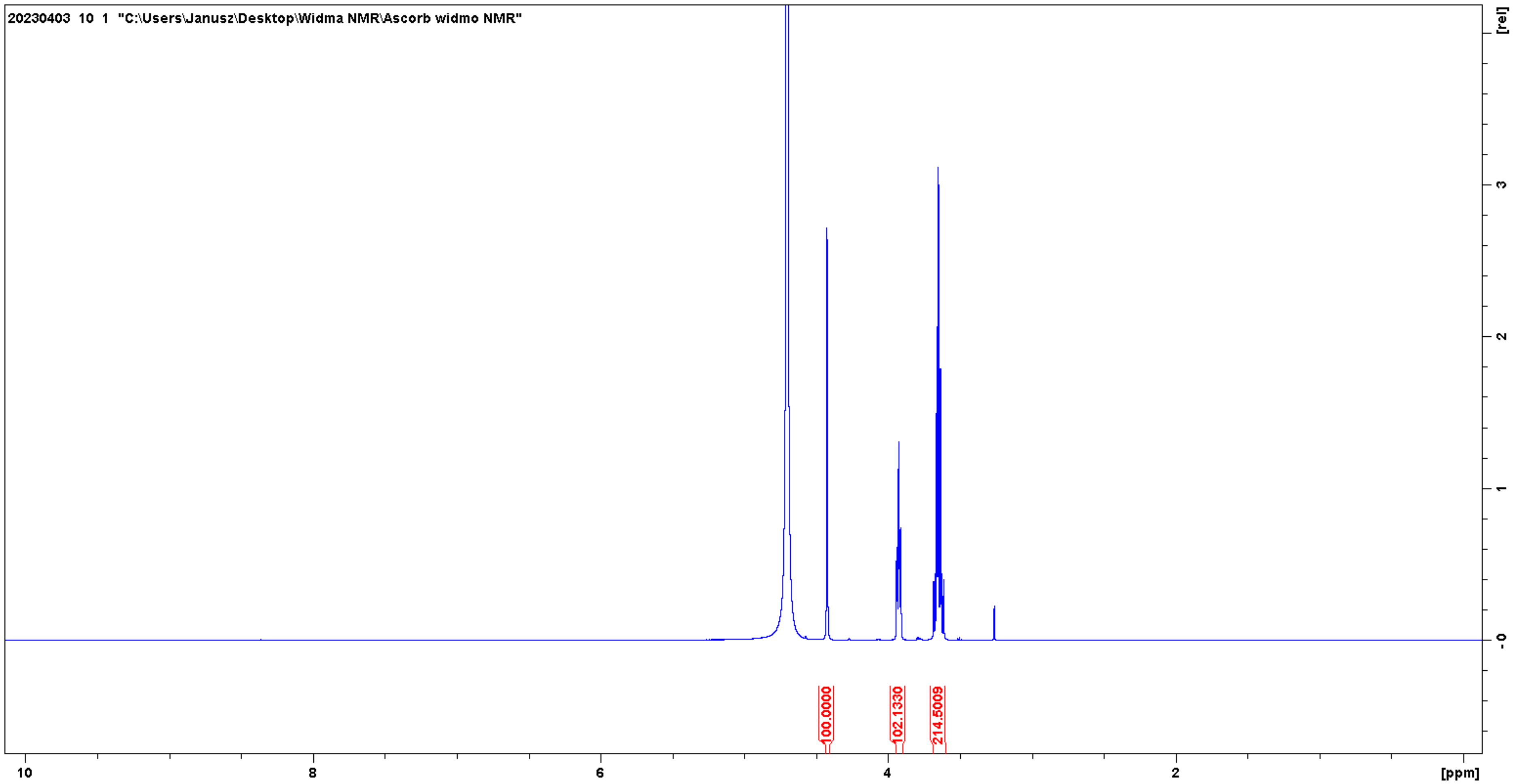

Figure S21. ^{1}H NMR spectrum (D_2O, 500 MHz) of hydrazinium L-ascorbate (**6**).

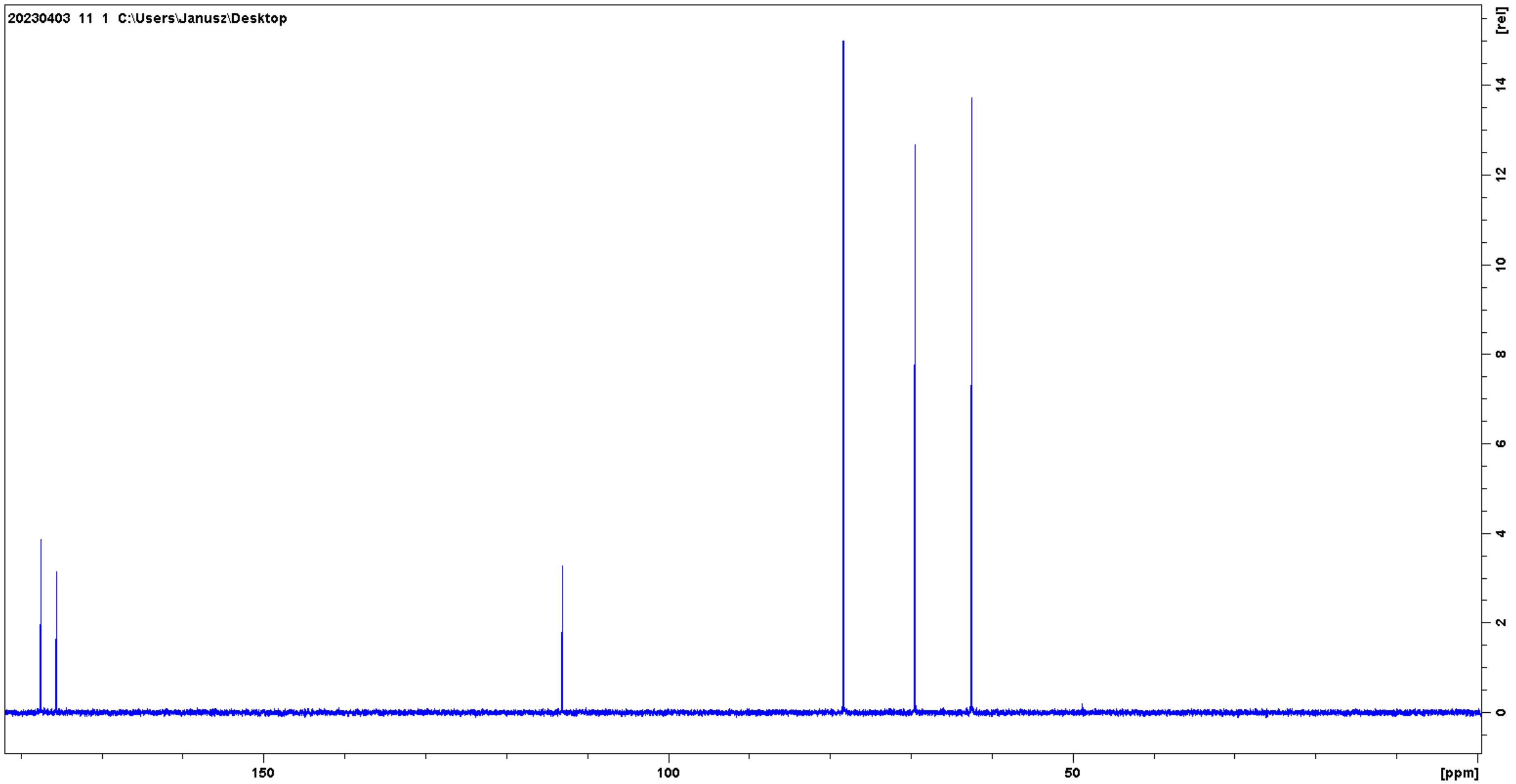

Figure S22. ^{13}C NMR spectrum (D_2O, 125 MHz) of hydrazinium L-ascorbate (**6**).

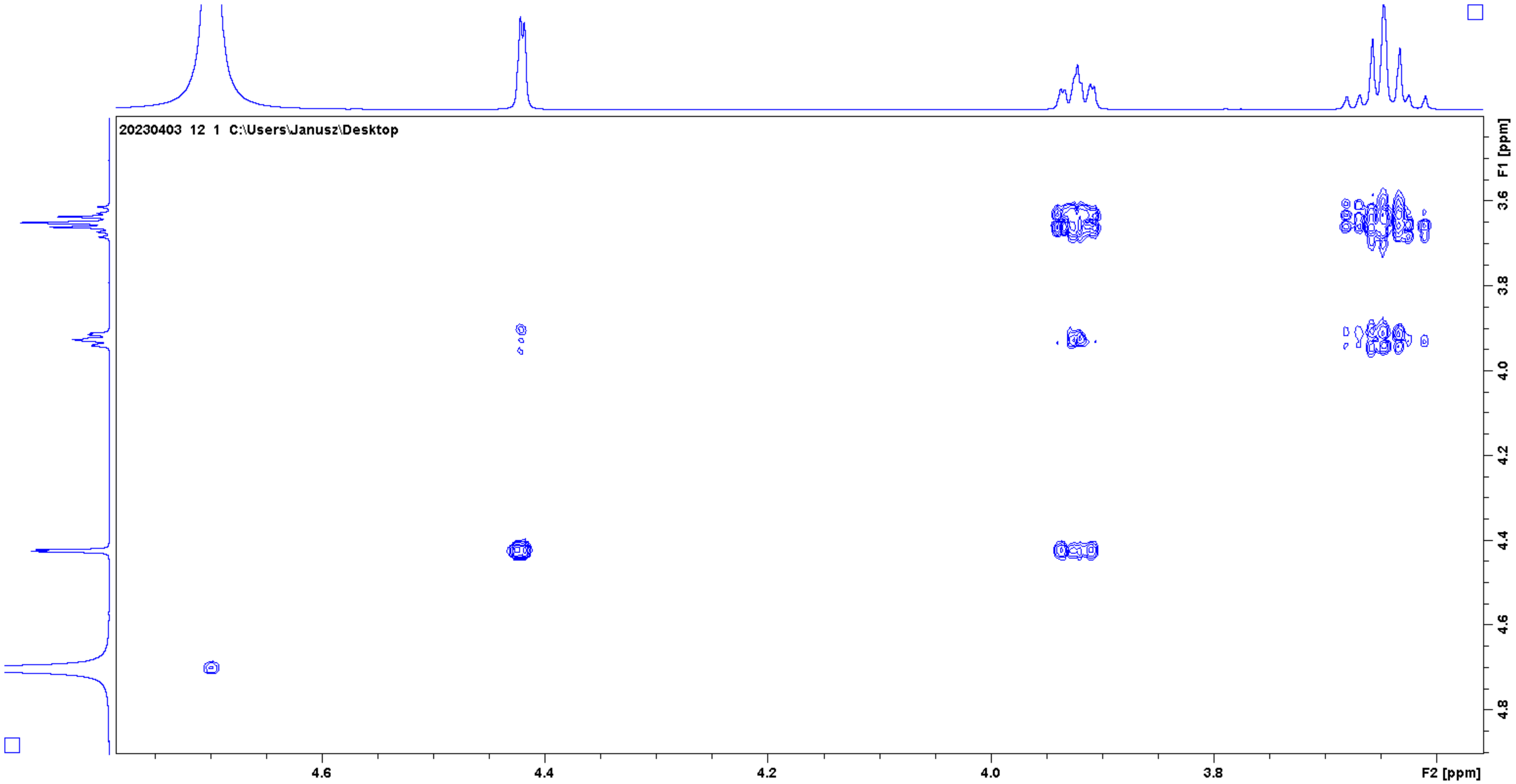

Figure S23. COSY spectrum of hydrazinium L-ascorbate (**6**).

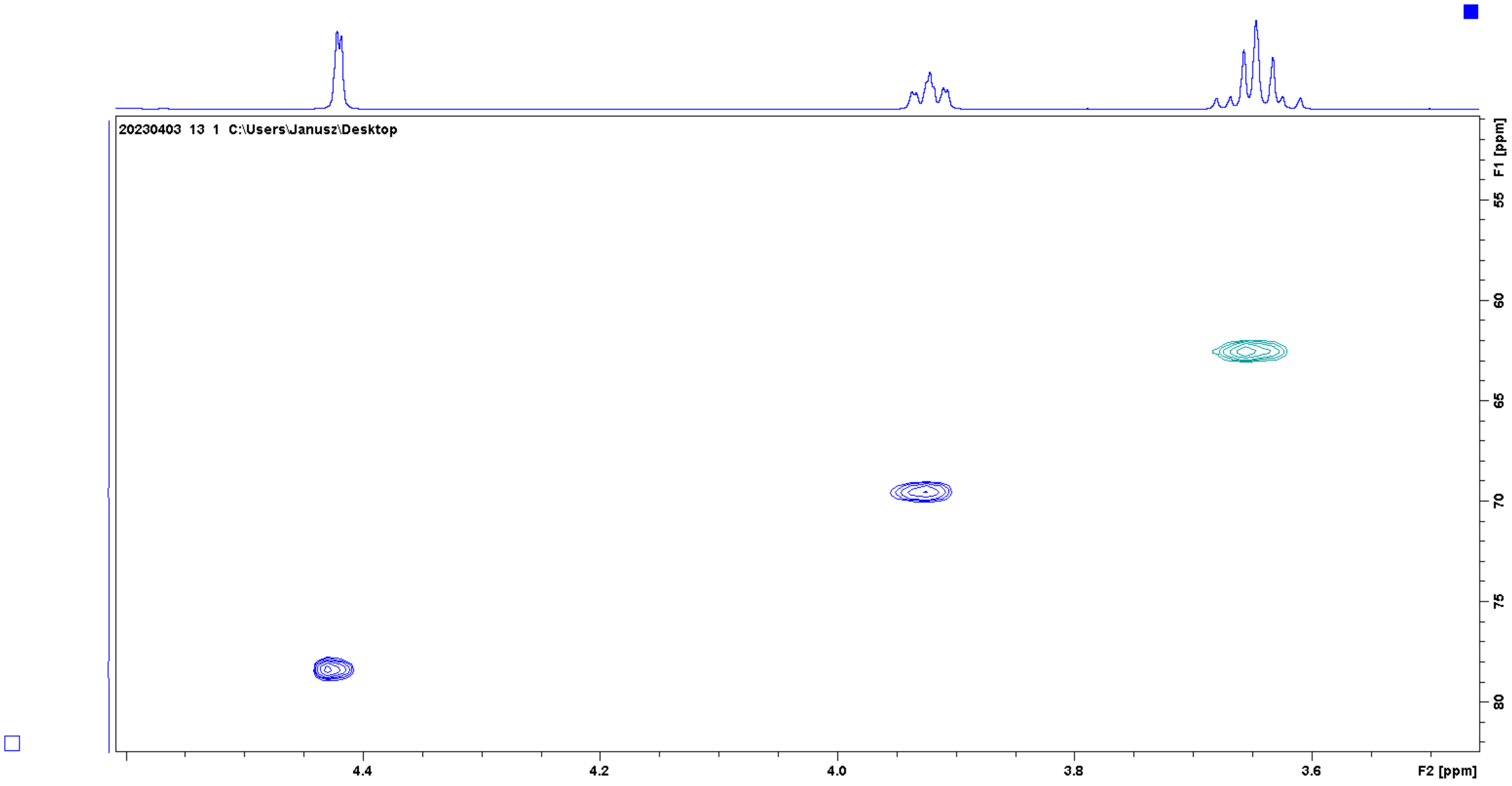

Figure S24. HSQC DEPT spectrum of hydrazinium L-ascorbate (**6**).

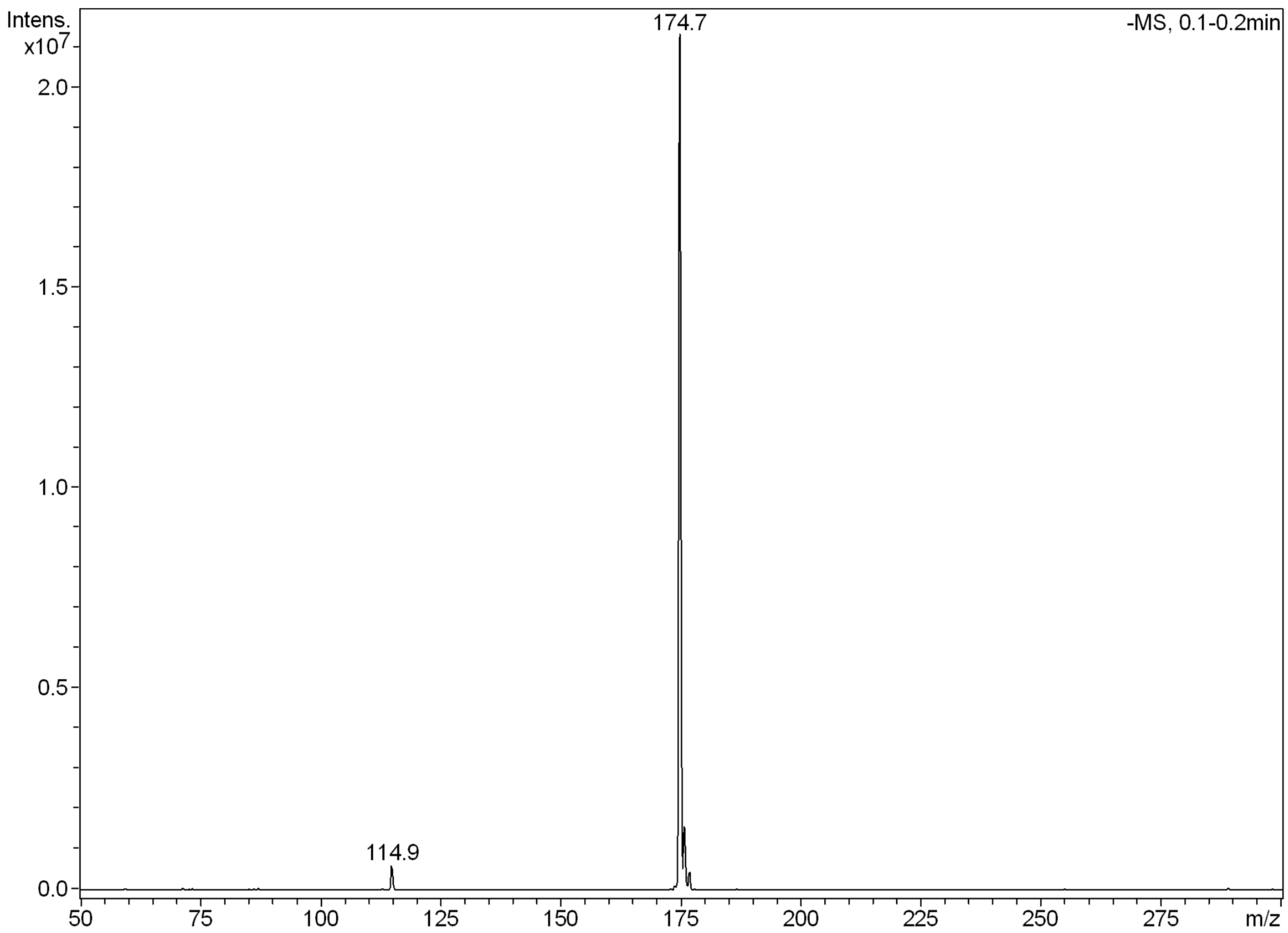

Figure S25. Negative ion mode ESI MS spectrum of **6**.

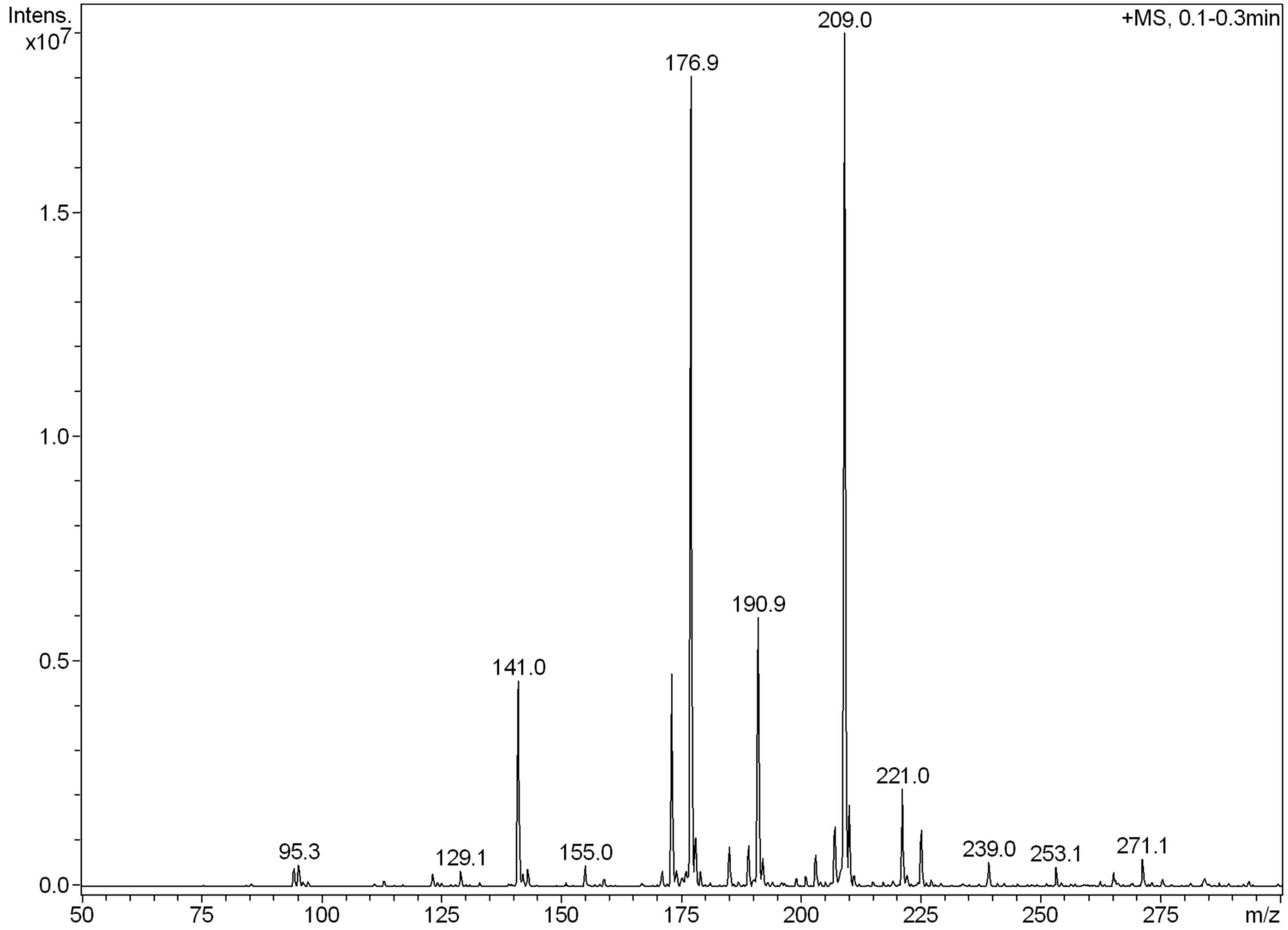

Figure S26. Positive ion mode ESI MS spectrum of **6**.

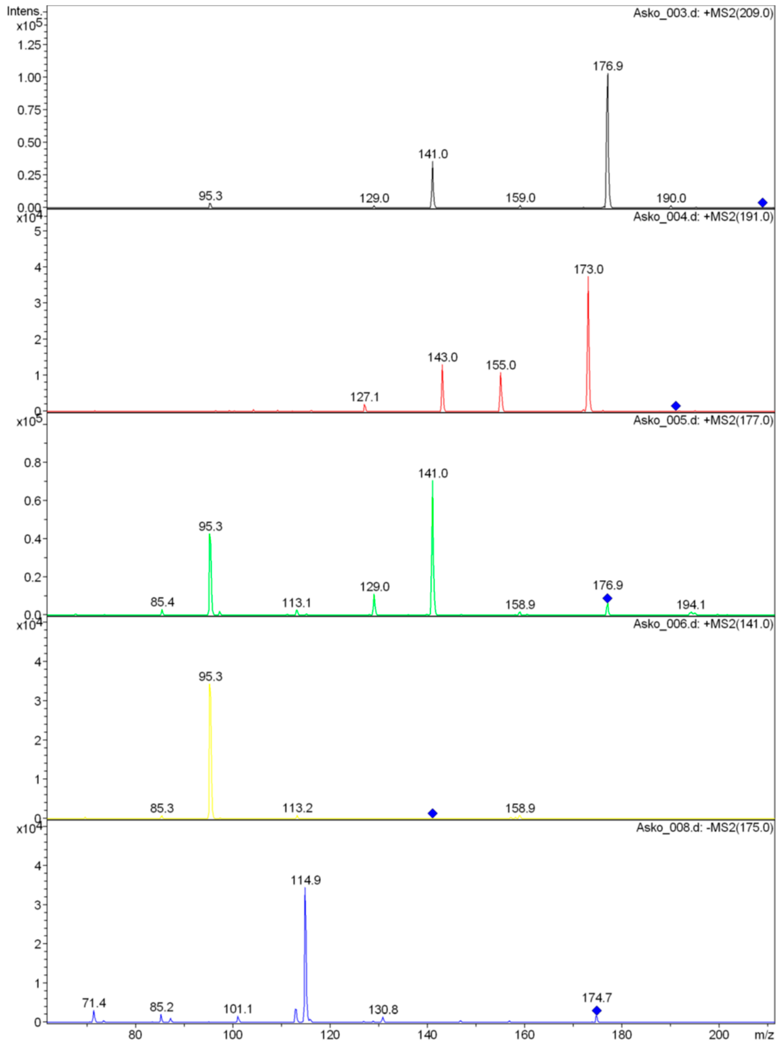

Figure S27. Positive ion mode MS-MS spectrum of **6**.

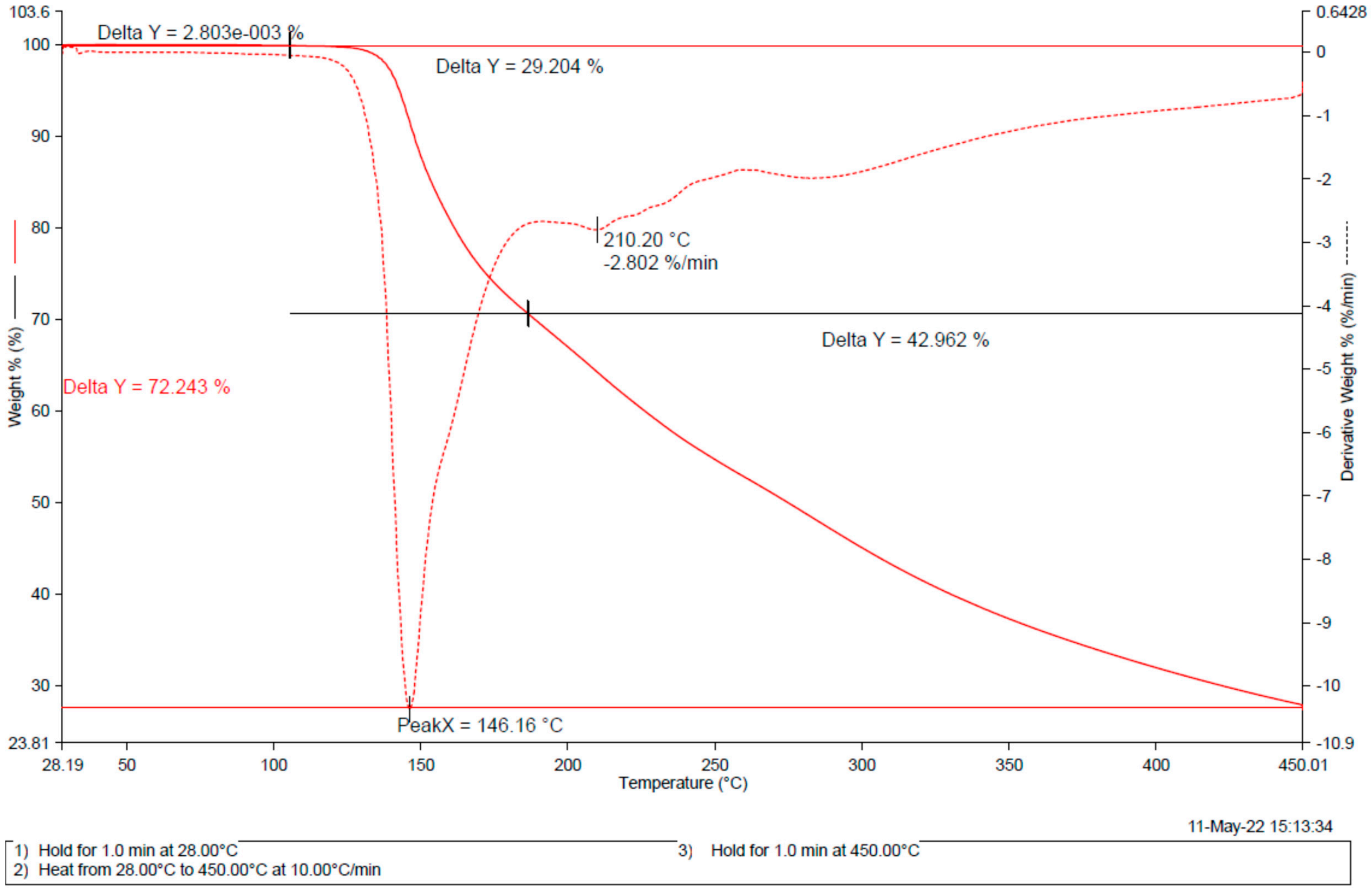

Figure S28. Thermogram (TG) and derivative weight loss (DTG) of **6**.

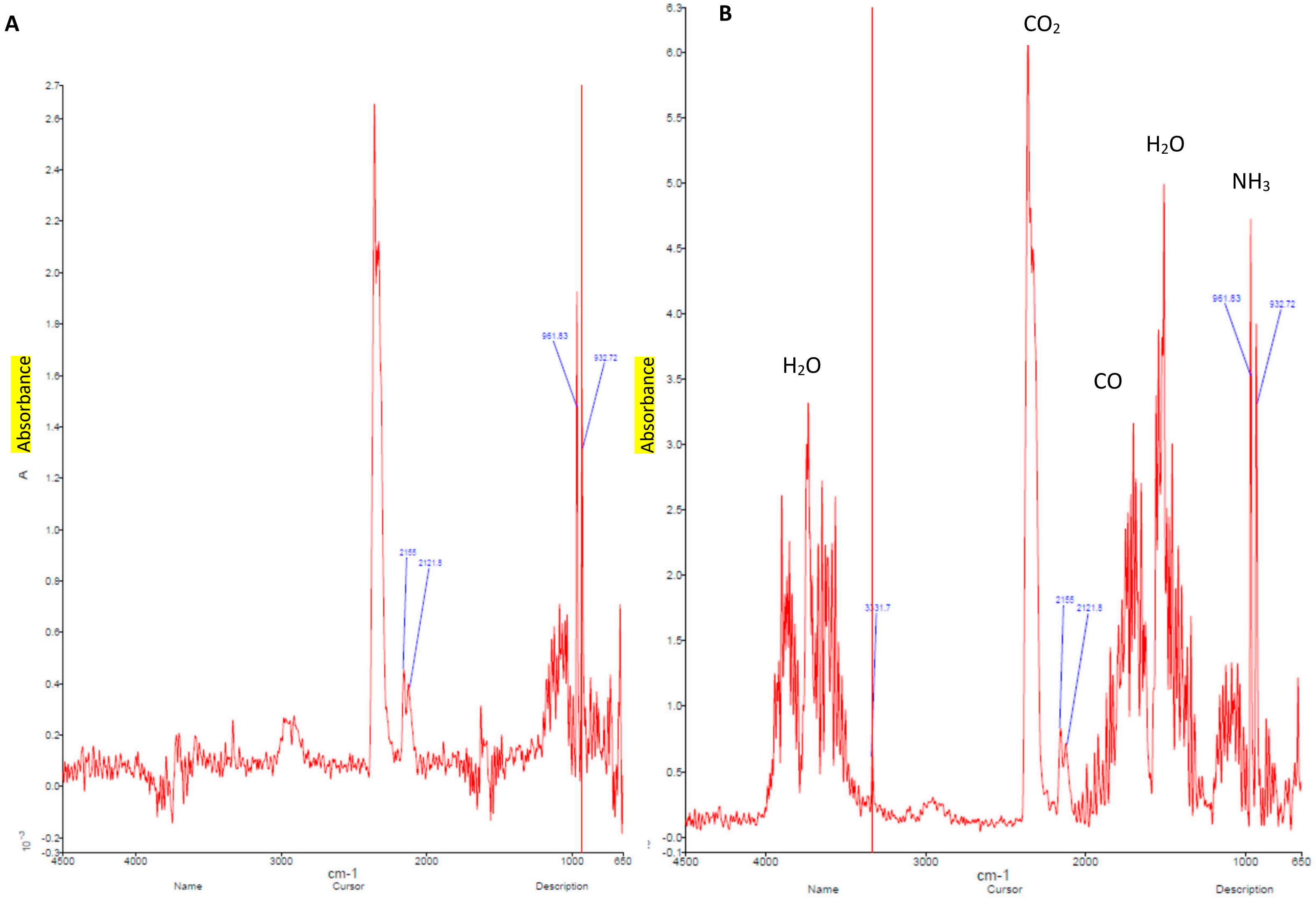

Figure S29. IR spectrum of gaseous products formed during thermal decomposition of compound **6** in: A 12.44 min, B 13.15 min.
